# Flower Bud Transcriptome Analysis of *Sapium sebiferum* (Linn.) Roxb. and Primary Investigation of Drought Induced Flowering: Pathway Construction and G-Quadruplex Prediction Based on Transcriptome

**DOI:** 10.1371/journal.pone.0118479

**Published:** 2015-03-04

**Authors:** Minglei Yang, Ying Wu, Shan Jin, Jinyan Hou, Yingji Mao, Wenbo Liu, Yangcheng Shen, Lifang Wu

**Affiliations:** 1 Key Laboratory of Ion Beam Bioengineering and Bioenergy Forest Research Center of State Forestry Administration, Hefei Institutes of Physical Science, Chinese Academy of Sciences, Hefei, Anhui, People’s Republic of China; 2 School of Life Science, University of Science and Technology of China, Hefei, Anhui, People’s Republic of China; 3 College of Food and Bioengineering, Henan University of Science and Technology, Luoyang, Henan, People’s Republic of China; 4 School of Life Science, Anhui University, Hefei, Anhui, People’s Republic of China; Nanjing Agricultural University, CHINA

## Abstract

*Sapium sebiferum* (Linn.) Roxb. (Chinese Tallow Tree) is a perennial woody tree and its seeds are rich in oil which hold great potential for biodiesel production. Despite a traditional woody oil plant, our understanding on *S. sebiferum* genetics and molecular biology remains scant. In this study, the first comprehensive transcriptome of *S. sebiferum* flower has been generated by sequencing and *de novo* assembly. A total of 149,342 unigenes were generated from raw reads, of which 24,289 unigenes were successfully matched to public database. A total of 61 MADS box genes and putative pathways involved in *S. sebiferum* flower development have been identified. Abiotic stress response network was also constructed in this work, where 2,686 unigenes are involved in the pathway. As for lipid biosynthesis, 161 unigenes have been identified in fatty acid (FA) and triacylglycerol (TAG) biosynthesis. Besides, the G-Quadruplexes in RNA of *S. sebiferum* also have been predicted. An interesting finding is that the stress-induced flowering was observed in *S. sebiferum* for the first time. According to the results of semi-quantitative PCR, expression tendencies of flowering-related genes, *GA1*, *AP2* and *CRY2*, accorded with stress-related genes, such as *GRX50435* and *PRXⅡ39562*. This transcriptome provides functional genomic information for further research of *S. sebiferum*, especially for the genetic engineering to shorten the juvenile period and improve yield by regulating flower development. It also offers a useful database for the research of other *Euphorbiaceae* family plants.

## Introduction

As global fossil energy crises and environmental deterioration get worse, the latest researches are more focused on bioenergy, a new potential substitutive energy. Traditional biodiesel is made from soybean, rapeseed, groundnut, sunflower, et al., which have potential danger of invasion into food-crop lands [1, 2]. Hence, oil seed plants such as *Jatropha curcas* and *S. sebiferum* have more advantages than traditional biodiesel crops because of their additional benefits of greening wastelands and increasing marginal land utilization.

*S. sebiferum* (Euphorbiaceae) is a perennial woody tree. It is native to eastern Asia and widely spread in China and Japan. In China, it also has a long history as a herbal medicine [3] and in candle production. Besides, the seeds of *S. sebiferum* can be used to produce cruid oil which contains oleic acid (15.12%), linoleic acid (31.60%), caproleic acid (2.70%), linolenic acid (44.19%) and palmitic acid (6.38%) [4]. The index of oil (cetane number, kinematic of viscosity and exhaust emission) meets the requirement of biodiesel production. Previous research indicated that chemical component of caproleic acid is 2t,4c-decadienoic acid (10:2Δ^2t,4c^_,_ stillingic acid), which belongs to conjugated fatty acids(CFA). Conjugated fatty acids (CFAs) are geometric isomers of polyunsaturated fatty acids with conjugated double bonds and have potential effects against cancer, obesity et al. [5].

*S. sebiferum* has great productive potential with desirable properties of biodiesel, which encourages plant biologists to modify the plants through genetic engineering. One efficient way to maximize oil productivity is to increase the numbers of flowers, especially the ratio of female flower to male flower. However, constraints arise due to the lack of basic biological knowledge of *S. sebiferum*. To date, most studies on *S. sebiferum* are involved in the population distribution as biological invasion plants, bioactive compounds and the evaluation on the physical-chemistry property of seed oil as biodiesel. Few basic biological and genetics information was found in the present literatures. So far, the only cognition on reproductive development was its androgynous sexual system, similar to plants of the *Cyperaceae* family. Therefore, a wide range of further research needs to be focused on *S. sebiferum*, especially in biochemistry and genetics. Due to the high heterozygosity [6] and massive genome(2n = 88, http://gjk.scib.ac.cn/chromosome/resultabs.asp?id=1385), it is more difficult to apply traditional genetic manipulation on *S. sebiferum*. However, next generation sequencing (NGS) methods offer a rapid and cost-effective way to obtain a large amount of genetic information. Thus, NGS, especially *de novo* mRNA sequencing (transcriptome), has been widely applied in non-model species without prior genome information. Huge genes or gene fragments from transcriptome can be annotated through multimodal comparative analysis with sequences of other organism in database, e.g. NCBI, Swiss-Prot, PlantGDB, Pytozome, TAIR, Gene Ontology, KEGG.

Extensive studies on flower development have revealed that an elaborate genetic network transduces external signal (vernalization, photoperiod) and integrates developmental signal (gibberellin and autonomous pathways) to promote transition from vegetative growth to reproductive growth [7]. Many key genes have been identified and functionally characterized by mutant analysis in *Arabidopsis thaliana*, in which a set of‘floral integrator’ genes (*AP1* and *SOC1*) integrate the outputs of the various pathways and directly activate floral meristem identity genes. Most of the genes involved in flowering transition and flower development belong to the family of MADS-box genes, which are usually the targets of genetic modification to regulate the flower development process to increase yield [8]. Besides, flowering in many plant species can be induced by stress condition, as reviewed by Kaede [9]. Although the exact mechanism underlying stress-induced flowering was not well studied, the genes involved in this process have been primarily investigated. The *FT*[9, 10], which functions in flowering, together with putative stress-related genes e.g. *GRX* [11], plays important roles in stress-induced flowering.

Guanine-rich nucleic acids can spontaneously fold into non-canonical DNA secondary structures named G-Quadruplexes. The formation of these advanced structures may interfere with expressional homeostasis via possible mechanisms associated with transcription, translation, splicing, telomere maintenance and DNA recombination [12]. RNA can also form various secondary and tertiary structures, such as duplexes, hairpins, quadruplexes and pseudo-knots. And it has been proved that stress response genes showed significantly difference *in vivo* RNA secondary structure from their unconstrained *in silico* predictions, which indicated these genes may have evolved to resist large conformational changes in order to maintain cell homeostasis [13].The prediction of G-Quadruplexes in transcriptome of *S. sebiferum* is helpful to further understand the function of gene secondary structure in *S. sebiferum*.

In this paper, the annotation of MADS box family based on *S. sebiferum* transcriptome was applied as it plays a critical role in flowering development. Putative pathways of lipid synthesis and abiotic stress also have been constructed based on EST data. Another study is about the prediction of G-Quadruplexes in transcriptome of *S. sebiferum*. These bioinformatics analyses provide a biological reference for agriculturally important gene operation and molecular breeding in *S. sebferum*.

## Results and Discussion

### Illumina sequencing and *de novo* assembly

**Fig 1:**
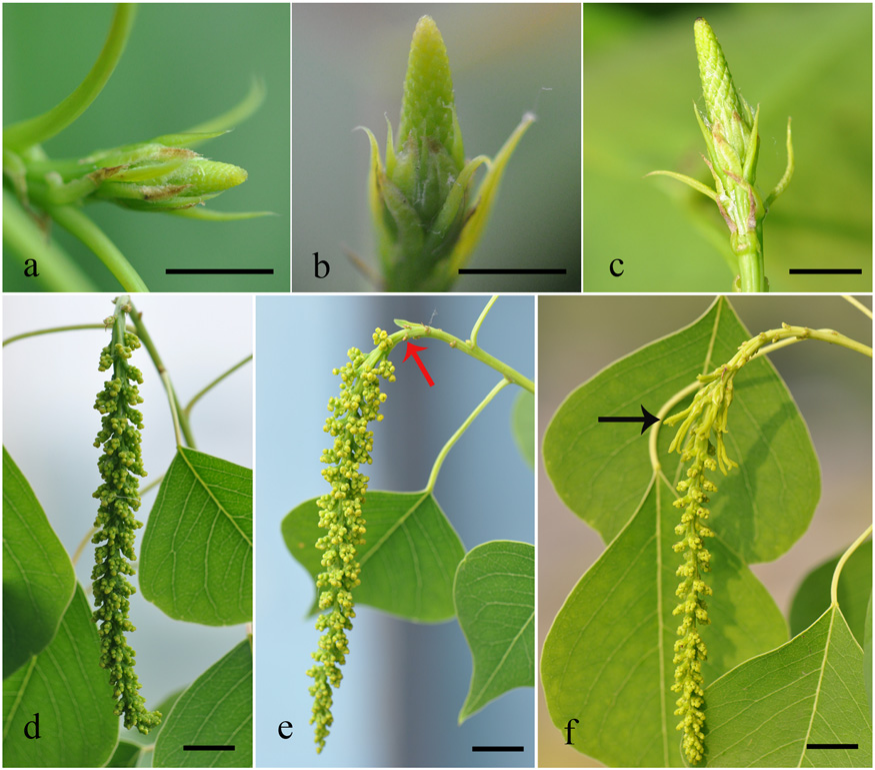
Flower development stages in *S. sebiferum*. *S. sebiferum* flower development can be divided into six fine stages: a. inflorescence bud swelling; b. inflorescence emergence; c. inflorescence expansion; d. staminate flower partially open with pistillate flower buds emergence; e. staminate flower fully open with pistillate flower buds expansion; f. pistillate flower fully open. Scale bar = 1 cm. Red arrow represents pistillate flower bud and black arrow represents pistillate flowers.

The systematical phenological observation of *S. sebiferum*, including vegetable growth and reproductive growth, has been carried out by Vikrant Jaryan et al. [14]. In our study, we divided *S. sebiferum* flower development into six stages (Fig. 1): inflorescence bud swelling (Fig. 1a); inflorescence emergence (Fig. 1b); inflorescence expansion (Fig. 1c); staminate flower partially open with pistillate flower buds emergence (Fig. 1d); staminate flower fully open with pistillate flower buds expansion (Fig. 1e); pistillate flower fully open (Fig. 1f).

**Fig 2:**
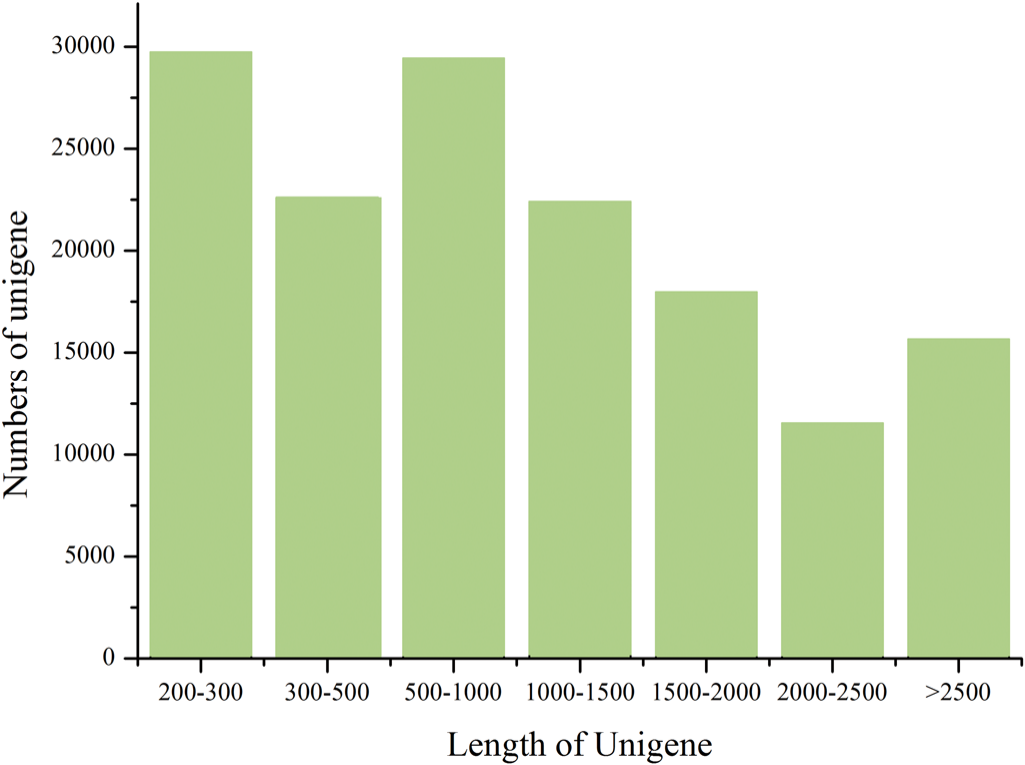
The distribution of the unigenes’ lengths. The horizontal and vertical axes show the size and the number of unigenes respectively.

Inflorescence (Fig. 1a, 1b, 1c), staminate flower buds (Fig. 1d) and pistillate flower buds (Fig. 1e) were collected respectively and immediately frozen in liquid nitrogen. Then the samples were applied for RNA extraction and Illumina paired-end sequencing by Berry Genomic Inc. A total of 64,663,388 raw sequencing reads were generated from the library. After removing low-quality reads, the remaining high-quality reads were used to assemble the transcriptome of *S. sebiferum* flower buds, and 129,434 unigenes were generated with an average length of 1,175 bp and a N50 of 1,857bp. The unigenes with the length more than 500bp accounted for about 64.95% (Fig. 2). The length of the longest unigene sequence was 9,651 base pairs (unigene 89867). To identify the function of the longest gene, BlastX against NCBI was applied and uncovered the conserved BEACH domain followed by a series of WD repeat, which is a common pattern in BEACH domain containing protein. Although the function of BEACH domain is still unknown, the WD domain containing protein has been reported to be involved in flowering or flower development [15]. That displayed the typical character of the transcriptome from flower tissue.

### Function annotation by searching against public database

To validate and annotate the assembled unigenes, all the unigenes were searched against NCBI nucleotide sequence database (NT), Non-redundant protein sequence database (NR) and Swiss-Prot protein database using BlastN and BlastX program with an E-value threshold of 1E-10.

**Fig 3:**
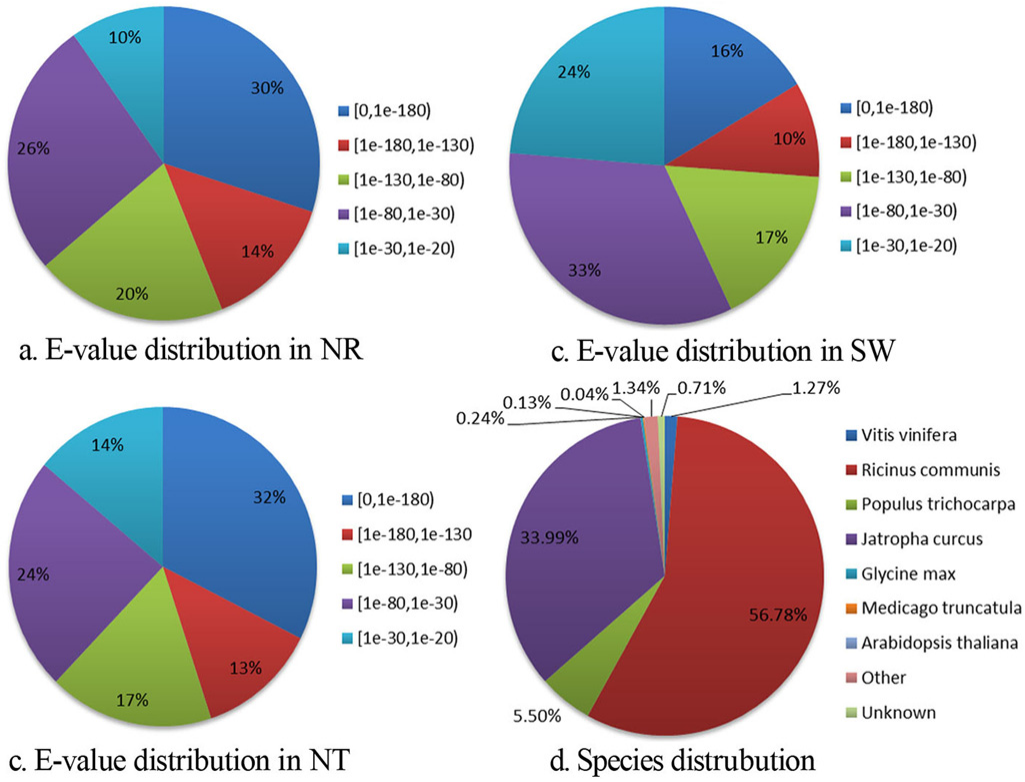
Characterization of searching the assembled unigenes against NR, NT and Swiss-Prot protein databases. a. E-value distribution of BLAST hits for the assembled unigenes in NR database; b.E-value distribution of BLAST hits for the assembled unigenes in NT; c. E-value distribution of BLAST hits for the assembled unigenes in Swiss-Prot database. d. Species distribution of top hits in NT database.

Among 129,434 unigenes, 85,738 (66.24%) were found to have significant similarity with corresponding unique genes in NT database, 82,144 (63.46%) with corresponding genes in NR database. Of all assembled unigenes, 53,382 (41.24%) were matched to unique protein accession numbers with significant identities in Swiss-Prot database. The E-value distribution of top hits revealed that 63% of the alignment sequences had significant homology (< 1E-80) to entries in NT database. As for blast against NR protein database and Swiss-Prot database, the corresponding E-value (< 1E-80) was 63.2% and 43%, respectively (Fig. 3).

Of all the unigenes, seventy unigenes whose FPKM were more than 6000 have the most abundant transcript in *S. sebiferum* flower buds (S1 Table). As flower buds proceeded into blooming state, transcription factors (TFs) associated with flowering were highly expressed including processing protein (PRP39) [16] and WD-repeat protein [15]. A highly expressed gene similar to *LHY* (*LATE ELONGATED HYPOCOTYL*) indicated that photoperiod played an important role in flowering. Other proteins involved in signal transduction such as auxin response factor and stress response protein were also predominantly expressed (S1 Table).

### Functional classification by GO and KEGG

**Fig 4:**
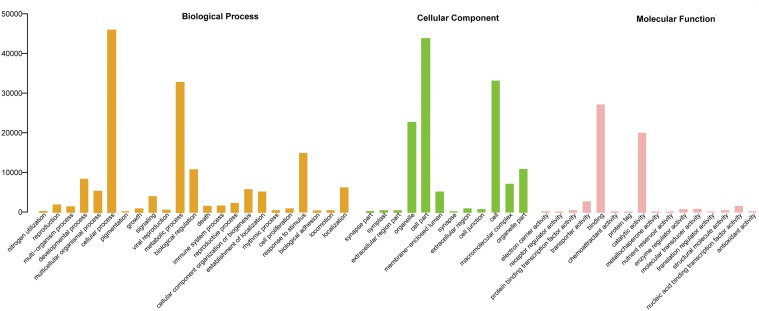
Gene Ontology (GO) classifications of assembled unigenes. 17,243 unigenes of *S.sebiferm* were assigned into the three main GO functional categories including biological process (45.61%), cellular component (38.107%) and molecular function (16.29%)

Gene Ontology (GO) is an international standardized gene function classification system, and it is useful to annotate and analyze the functions of a large number of genes in any organism. 17,243 unigenes of *S. sebiferum* were assigned into three main GO functional categories including biological process (45.61%), cellular component (38.107%) and molecular function (16.29%). They were then categorized into 50 sub-categories (Fig. 4).

The Kyoto Encyclopedia of Genes and Genomes (KEGG) Pathway is a collection of manually drawn pathway maps that display the molecular interaction and reaction networks. The pathway-based analysis is helpful to further understand the biological functions and interactions of genes. All the unigenes of *S. sebiferum* were analyzed in KEGG pathway database. 6,367 unigenges were assigned to 5 main categories including 105 KEGG pathways (S2 Table), and metabolism related unigenes (4,852, 76%) were the majority in all categories.

### MADS box gene identification and phylogeny tree construction

**Fig 5:**
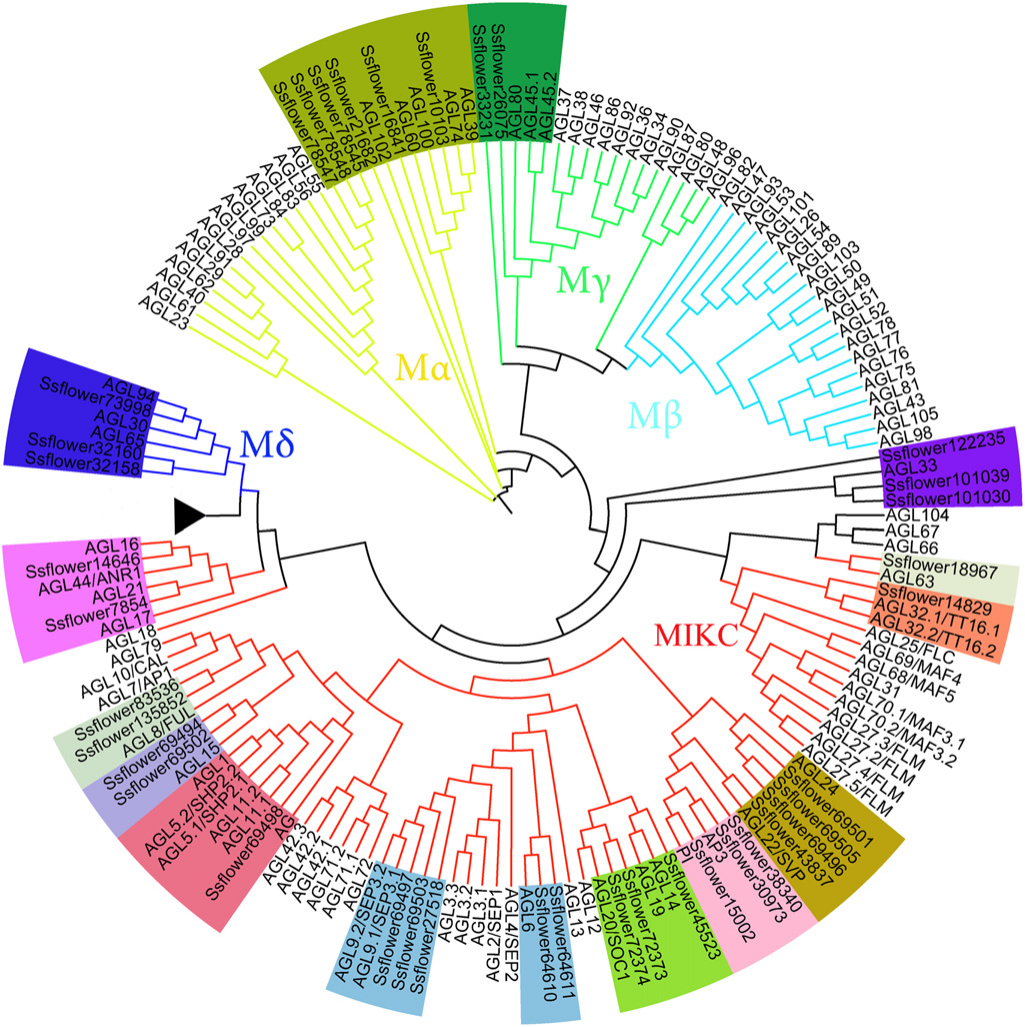
Phylogenetic Analysis of MADS-Box Containing Proteins of *S. sebiferum*. Red branches, MIKC; Blue branches, Mδ; Cyan branches, Mβ; Green, Mγ; gold, Mα

It has been noted previously that Hidden Markov model (HMM) was three times sensitive than Blast methods and more efficient to detect remote homologues [17]. According to the manual of HMM 3.0, a profile was built out of *Arabidopsis* MADS box gene family, some of which were fully analyzed in genetics and were suitable to speculate the possible biological functions of their homologues (see Methods). The profile then was used to search the transcriptome and 61 MADS box genes were successfully identified with the E-value < 0.01. Thirty-eight sequences of the 61 MADS box genes whose length was more than 100 amino acids were then aligned with *Arabidopsis* MADS box domain sequences to construct a phylogenetic tree using MEGA 5.0 [18, 19]. According to their relationship with Arabidopsis MADS box genes, twenty four sequences were unambiguously classified as MIKC type II MADS-box genes, while the rest were grouped into Mα, Mγ, Mδ and AGL33, respectively. No counterpart of Arabidopsis Mβ group was found in *S. sebiferum* (Fig. 5). The literature showed more than one hundred MADS box genes were identified from *Populus trichocarpa* [20], which covered all the five types (Mα, Mβ, Mγ, Mδ and MIKC). And the MIKC is also the dominant type, accounting for more than half of the MADS box genes. The above analysis indicated that there is a big difference in the number and the type of MADS box genes between *P. trichocarpa* and *S. sebiferum*. Besides the species character, an important reason was that the prediction was based on whole genome analysis in *P. trichocarpa*. With the further accomplishment of *S. sebiferum* genome sequencing, more MADS box genes will be identified.

### Putative pathway of flower development and drought induced flowering

Since *S. sebiferum* is a perennial tree with long vegetative growth, it is meaningful to bring forward the tree’s flowering time to increase fruit yield. Based on the understanding of flower development, molecular breeding biologists can carry out ingenious molecular designs to change the flower development process to increase yield. For example, Jiang Ke [8] improved the yield of tomato by balancing the mutual antagonistic genes *SELF FLOWER TRUSS* (*SFT*) and *SELF PRUNNING* (*SP*). *SFT*, which is an ortholog of *FT* (*FLOWE LOCUS T*) in Arabidopsis, promotes flowering. Functionally contrary to the former, *SP*, homologous to *FLC* (*FLOWER LOCUS C*) in *Arabidopsis*, is a suppressor of flowering. The balance between these two antagonistic genes can trigger the flowering pathway to increase yield in tomato. *S. sebiferum* responds to various external signals (photoperiod, vernalization and ambient temperature) and internal signals (autonomous, age, gibberellins) to transform from vegetative growth to reproductive growth through elaborate genetic network [21]. These inductive signals must initially inhibit the expression of several flowering repressor genes, then turn on the floral transition integration genes (*AP1* or *LFY*).

*S. sebiferum* begins to bloom in April (Hefei, China), when the inductive photoperiod signal is perceived by green leaves and it results in the accumulation of *FT* regulated by *CO*, together with transcription factor *NF-YB* and *NF-YC*. The expression of *FT* determines the timing of flowering, therefore, its transcription is tightly regulated by many transcription factors as well as chromatin state of *FT*. The capability of *CO* to induce *FT* expression is counteracted by several repressors of *FT* through different pathways, which include RAV transcription factors *TEMPRANILLO1* and 2 (*TEM1* and *TEM2*) [22], the APETALA2 domain proteins *TARGET OF EAT 1–3* (*TOE1–3*), *SCHLAFMUTZE* (*SMZ*) and *SCHNARCHZAPFEN* (*SNZ*) [23–26] as well as the MADS domain factor *FLOWERING LOCUS C* (*FLC*).

**Fig 6:**
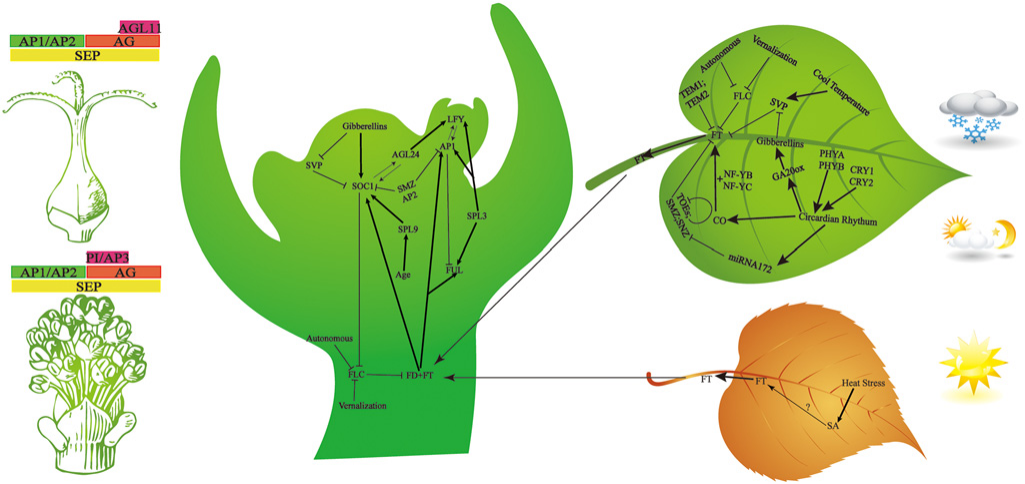
Putative pathway of flower development in *S. sebiferum*. External (vernalization, photoperiod) and internal (autonomous, age, gibberellins) signals integrate into elaborate genetic network to regulate flowering. The genes components of the genetic network have been discussed in text.

The FT protein is transported through the vasculature to the apex [27], where it acts as an effective floral inducer and triggers the floral transition network. There, FT physically interacts with the bZIP transcription factor FD [28] and they coordinately up-regulate the MADS-box genes *SOC1*, *AP1* and *FUL* [29]. *AP1* acts as a hub in floral development pathway and plays a cardinal role in floral meristem development, which is also conserved in angiosperm [21]. Finally, all of the floral pathway integrator and floral identity genes work together and trigger the floral organ development to form *S. sebiferum* reproductive organ. The genes involved in flowering pathway (shown in Fig. 6) were identified through blast locally and were showed in S3 Table.

**Fig 7:**
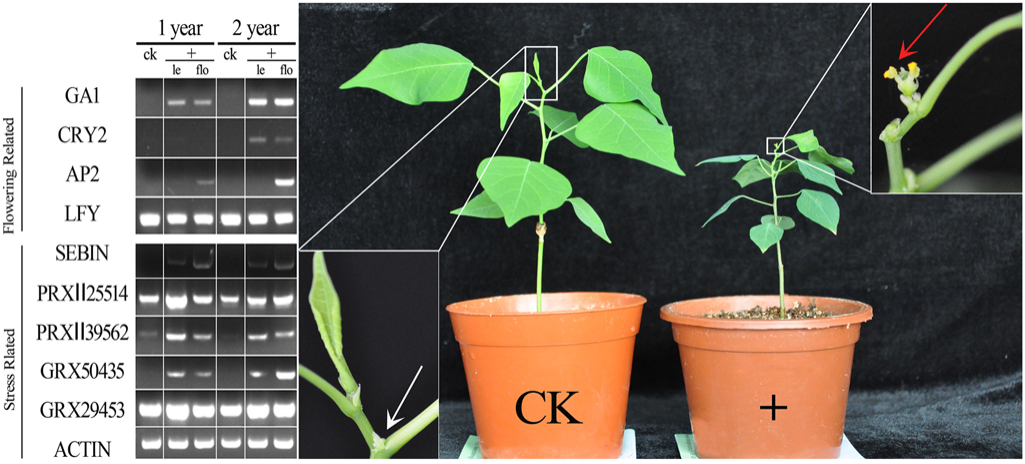
Drought induced flowering (right) and analysis of the expression of related genes by semi-quantitative PCR (left). Under drought stress, one-year-old seedling (+ in right) began to bloom (red arrow indicating the staminate flowers of *S. sebiferum*) and normal control (CK at the left) performed no flower or flower bud on the shoot apex (white arrow). Besides the difference of flower, the precocious-flowering seedling had distinct phonotype of senescence, such as dark leaf color and withered leaf tips. The expression of genes related with flowering and drought stress has been analyzed by semi-quantity PCR, and detailed discussion has been shown in text. le, leaves tissue; flo, flower tissue.

There was an interesting phenomenon observed in *S. sebiferum* for the first time by our lab. Under natural condition, *S. sebiferum* would bloom in three to five years after seeded; however, when one-year-old seedlings of *S. sebiferum* were exposed to drought conditions, the seedlings would precociously bloom (Fig. 7 right). To further analyze the possible mechanism, the putative key genes which may function in stress-induced flowering [10] have been investigated through semi-quantitative PCR (Fig. 7 left). The one-year-old and two-year-old seedlings under drought stress have been induced successfully to bloom, with ratio (induced-flowering plants in total plants) of 63% and 73% respectively.

The results of semi-quantitative PCR indicated that the expression tendencies of flowering-related genes, *GA1*, *AP2* and *CRY2* accorded with stress-related genes, such as *GRX50435* and *PRXⅡ39562*. Gibberellin is a factor of flowering initiation through regulation of *FT* [30](Fig. 6). *GA1*, ortholog of *AtGA1* (Ent-copalyl diphosphate synthase), plays an important role in gibberellin biosynthesis. The reaction catalyzed by *GA1* can be seen as the first committed step in gibberellin biosynthesis. According to semi-quantitative PCR analysis, *GA1* exhibited higher expression than control group, indicating that gibberellin may be functional in stress-induced flowering. It agreed with the common view of flowering biology of *Arabidopsis thaliana* [31], but was against fruits trees [32]. Expression of *AP2* was limited in the flower tissues of both one and two years old seedlings and it initiated the development of sepal in MADS model (Fig. 6) [33]. *CRY2* gene was only detected in two-years-old seedling in leaf and flower, and we inferred that *CRY2* was the downstream gene of age-related pathway. Besides, *LFY* showed constitutive expression pattern both in leaf and flower during vegetable or reproductive growth, which is consistent with previous study [34].

Under stress conditions, ROS (Reactive Oxygen Species) will burst out immediately. Plants have to regulate the expression of *SOD* (Superoxide dismutase), *PRX*s (Peroxiredoxins), *GRX*s (Glutaredoxins) et al., which eliminate the possible damage of ROS and maintain the homeostasis of redox in cell. Interestingly, the redox can regulate the expression of MADS box gene *AG* to control the flower development [11, 35]. It can be inferred that PRX, GRX, and SOD may play important roles in flowering under stress. The semi-quantitative PCR results showed that the expression tendencies of *GRX50435*, *PRXⅡ39562* and *SEBIN* accorded with the expression of *AP2* and *GA1*, indicating the relation between florescence and redox homeostasis. It was worth mentioning that *SEBIN*, a new member of *RIP* (Ribosome Inactivation Protein) family, was identified in transcriptome. The study of ricin (RIP in *Ricinus communis* L.) in mice has revealed that ricin induces generation of ROS and altered cellular redox state [36]. As shown in Fig. 7, *SEBIN* expression was detected both in two kinds of flowering plants. Therefore, we inferred that *SEBIN* in *S. sebiferum* may also have the function of promoting flowering like *GRX* [11].

Stress induced flowering was not a peculiarity of *S. sebiferum*, but a common feature in plant kingdom. This phenomena also has been found in *Pharbitis nil* and *Lemna paucicostata* through poor nutrition, as well as *Arabidopsis thaliana* under ultraviolet C band exposure [37]. In *P. nil*, it has been noted that the treatment with salicylic acid (SA) prior to stress induction enhanced the flower-induced effect of poor nutrition stress [38]. Salicylic acid in *S. sebiferum* may also share the same mechanism of stress-induced flowering. Besides, the younger the leaves of *P. nil* were, the more possibly stress-induced flowering would take place [9], which was consistent with our observation in *S. sebiferum*. The uncovering of stress-induced flowering in *S. sebiferum* provides a possible way to shorten the vegetable growth. That will be significant useful in genetic research and breeding.

### Putative genes involved in fatty acid biosynthesis

Genetic manipulation to improve oil content attracts many scientists’ endeavor. Understanding the genetic regulation of lipid biosynthesis in *S. sebiferum* will contribute to genetic improvement of *S. sebiferum*. To date, there is no EST database from seeds. Since primary metabolism, e.g. lipid synthesis, is conserved in flower tissue and seed, we constructed the lipid biosynthesis pathway.

**Fig 8:**
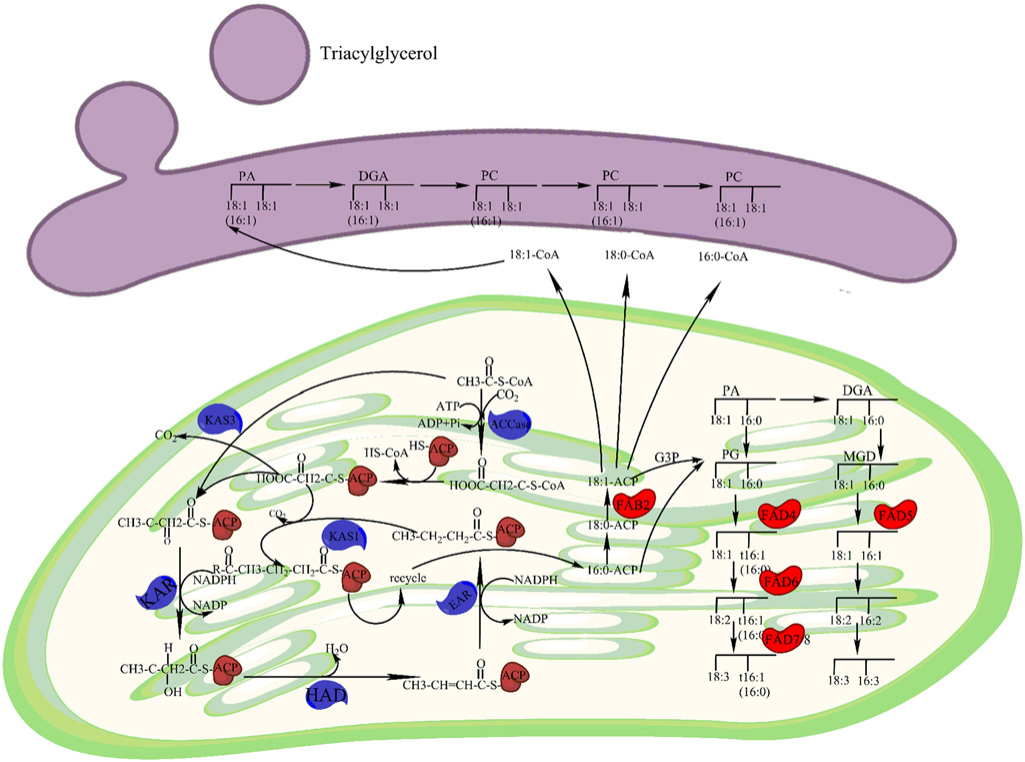
*De novo* fatty acid biosynthesis. Identified enzymes include ACCase, acetyl-CoA carboxylase carboxyl transferase (EC:6.4.1.2); KAS, 3-Ketoacyl ACP synthase (KASII, EC:2.3.1.179; KAS III, EC: 2.3.1.180); KAR, 3-Ketoacyl ACP reductase (EC:1.1.1.100); HAD, 3R-hydroxymyristoyl ACP dehydrase (EC:4.2.1.-); EAR, enoyl-ACP reductase I (EC:1.3.1.9); FATA/B, fatty acyl-ACP thioesterase A/B (EC:3.1.2.14 3.1.2.-); DGAT, diacylglycerol O-acyltransferase (EC:2.3.1.20); PDAT, phospholipid: diacylglycerol acyltransferase (EC:2.3.1.158); Lipid substrates are abbreviated: 16:0, palmitic acid; 18:0, stearic acid; 18:1, oleic acid; 18:2, linoleic acid.

Lipid biosynthesis involves *de novo* synthesis of saturated fatty acid, desaturation of fatty acid and synthesis of triacylglycerol (Fig. 8). The enzymes involved in *de novo* synthesis of fatty acid are listed in Table 1. ACCase (acetyl-CoA carboxylase carboxyl transferase) catalyzes fatty acid synthesis from the carboxylation of acetyl-CoA to malonyl-CoA, then CoA is exchanged in a subsequent reaction by acyl carrier protein (ACP) and proceeds downstream reaction as showed in Fig. 8. Fatty acid desaturases (FADs) play a central role in desaturation of fatty acid and all the FAD genes from the EST has been collected in S4 Table. Packaging nascent fatty acids into triacylglycerols (TAGs) requires the catalyzation of DAGT (Acyl-CoA:Diacylglycerol Acyltransferase) and PDAT (Phospholipid:Diacylglycerol Acyltransferase). The two kinds of genes identified in EST were shown in S5 Table. Besides, studies indicate that CFAs (the conjugated fatty acids) have various effects, including antitumor, anti-obese, anti-atherogenic and anti-diabetic activities [5]. Stillingic acid (2t,4c-decadienoic acid, 10:2Δ^2t,4c^) is uniquely enriched in *S. sebiferum* seed oils, which is one of the conjugated fatty acids [4]. FAD may be the critical enzyme in stillingic acid metabolism network.

**Table 1:** Enzymes involved in *de novo* fatty acid biosynthesis.

ID	Enzyme	Top Hit Sequence	Species	E-value
103616	acetyl-CoA carboxylase carboxyl transferase	KDP24495.1	*Jatropha curcas*	0.0
74489	3-Ketoacyl ACP reductase	AGT95889.1	*Vernicia fordii]*	2e-113
84018	beta-ketoacyl-ACP synthase II	ABJ90469.2	*Jatropha curcas*	0.0
53162	beta-hydroxyacyl-ACP dehydratase	HM244408.1	*Hevea brasiliensis*	0.0
59083	enoyl-ACP reductase	XM002515380.1	*Ricinus communis*	0.0

### Putative network for abiotic stress response

As sessile organisms, *S. sebiferum* is subject to stress, such as drought, cold, high temperature in its whole growth circle. Understanding the molecular mechanism of responses to abiotic stress is very important as it helps to improve stress tolerance and productivity of the plants.

**Fig 9:**
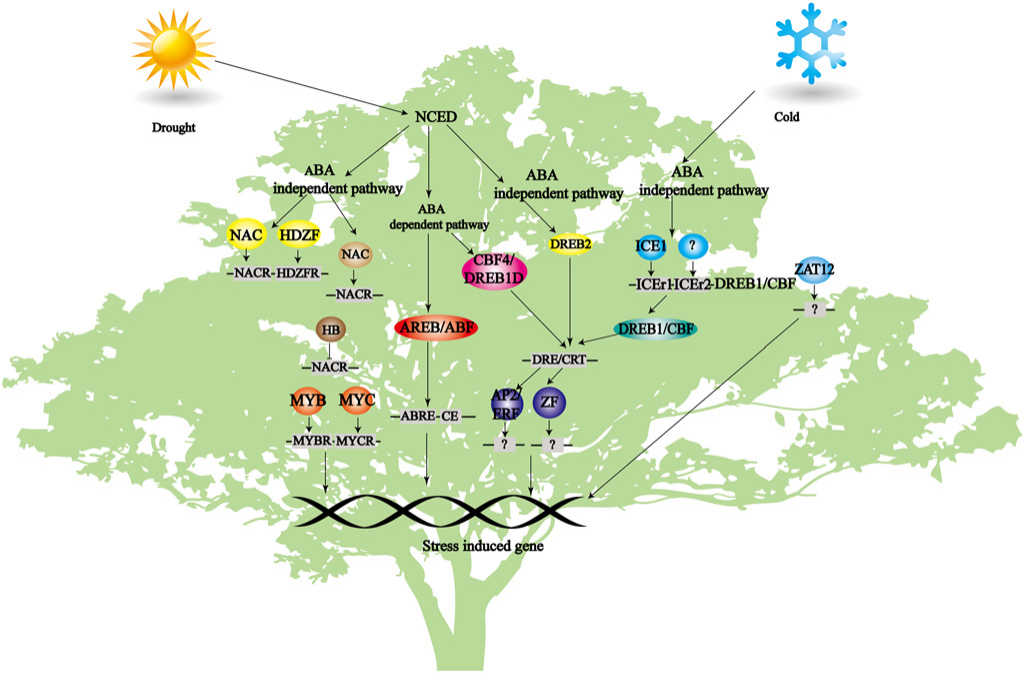
Stress response genetic network in *S. sebiferum*. ABA-dependent and ABA-independent pathway work together to response drought and cold. Identified transcription factors included *MYC*, *MYB*, *NAC* and *DREB*.

Based on the previous study, conserved stress-response genetic network underlying *S. sebiferum* has been constructed (Fig. 9). The first step in a signal transduction pathway is the perception of a signal, which is almost immediately followed by the generation of secondary signals, such as Ca^2+^, ROS. In spite of recent accumulation of knowledge about abiotic stress, the sensors involved in abiotic stress were still unknown. The work in simpler organisms such as *Synechocystis* and *Bacilus subtilis* indicated that transmembrane two-component systems have the potential role in transducing abiotic stress. Transmembrane two-component histidine kinases HIK33 (*Synechocystis*) and DesK (*B. subtilis*) have been suggested as thermosensors [39, 40]. Interestingly, the Arabidopsis histidine kinase Cre1, which functions as a cytokinin receptor, was observed to function as a sensor for changes in turgor pressure when expressed in yeast [41]. About 86 homolog genes have also been annotated by locally BLASTP against histidine kinase of *Arabidopsis thaliana* (accession number AEC05507).

It has been postulated that cold is sensed when changes in membrane fluidity [42] and cytoskeletal reorganization [43] affect calcium channels. Calcium-dependent protein kinases (CDPKs) are one of the largest subfamilies of plant protein kinases whose transcript induction has been observed in response to diverse abiotic stimuli. There are 500 CDPK homolog genes (E-value < 1E-8) annotated in transcriptome of *S. sebiferum*. Like CDPKs, MAPK cascades also function during abiotic stress signaling in plants [44] and are in large numbers (1,353 homolog genes with E-value <1E-3) in *S. sebiferum*.

ABA is an important plant hormone that regulates various aspects of plant development and plays a critical role in the regulation of water balance and osmotic stress tolerance. The rate limiting enzyme involved in ABA biosynthesis is 9-cis epoxycarotenoid dioxygenase (NCED), which cleaves neoxanthin into xanthonin [45] and is upregulated by osmotic stress [45, 46]. Transcription of *ABA1* [47], *AAO3* [48] and *ABA3* [49], which participate in converting ABA aldehyde to ABA, is also upregulated by osmotic stress. The homolog genes of *ABA1* and *AAO3* were identified by locally blast against transcriptome of *S. sebiferum* (Table 2).

**Table 2:** Main orthologs involved in abotic stress response.

Gene	Species	Top Hit Sequence	Cutoff of E-value	Numbers
Histidine kinase	Arabidopsis thaliana	AEC05507.1	1e-3	85
NCED6	Arabidopsis thaliana	NP_189064.1	1e-3	7
ABA1	Arabidopsis thaliana	AED98292.1	1e-3	32
MAPK	Glycine max	NP_001238209.1	1e-3	1354
CDPK	Populus tomentosa	AGM20664.1	1e-3	500
MYB	Arabidopsis thaliana	AT1G06180.1	1e-3	389
NAC(RD26)	Arabidopsis thaliana	AEE85335.1	1e-3	198
DREB	Glycine max	AAP83131	1e-3	121

Most ABA-inducible genes in ABA dependent pathway contain a conserved, 8 bp *cis*-acting sequence, designated as abscisic acid responsive element (ABRE, PyACGTGG/TC), with ACGT core in their promoter regions [50]. The most common and widely reported ABA regulated genes are the LEA or LEA like genes [51]. However, several stress-inducible genes, such as *RD29A*(responsive to dehydration29A)/*LTI78* (low-temperature induced78)/*COR78*(cold-regulated78), *RD17*(cor47), *KIN1*(cold-inducible1), are known to be only regulated by dehydration and cold stress in ABA-independent signaling pathways [51]. The promoters of ABA-independent-induced genes contain a *cis*-acting element, termed as dehydration-responsive element/cold-responsive element or C-repeat (DRE/CRT), which is essential for their function [52].

Various transcript elements(TEs) bind to certain motif of their target genes and regulate their expression, for example, the bZIP factors AREB/ABF are binding to ABREs, MYC/MYB proteins are binding to MYCRS and MYBRS; the DREB proteins activate the stress response through DREs in ABA-independent manner [51]. The TEs of NAC play a role through NACRs motif in the promoter region of target genes, such as *ERD1* [53, 54].

### Identification of G-Quadruplex formation

There are numerous mechanisms that regulate gene expression either at DNA level (methylation level of cytosine, promoter, enhancer, inhibitor and other *cis*-elements) or RNA level (e.g. mRNA processing). At RNA level, secondary structures formed in 5’- and 3’-UTRs can also serve as regulatory elements. Ding et al. [13] applied structure-seq to present the first *in vivo* genome-wide profiling of RNA secondary structure in *Arabidopsis thaliana*. The results indicated that stress-response RNAs may be more plastic, changing their structure in response to changing cellular conditions.

**Fig 10:**
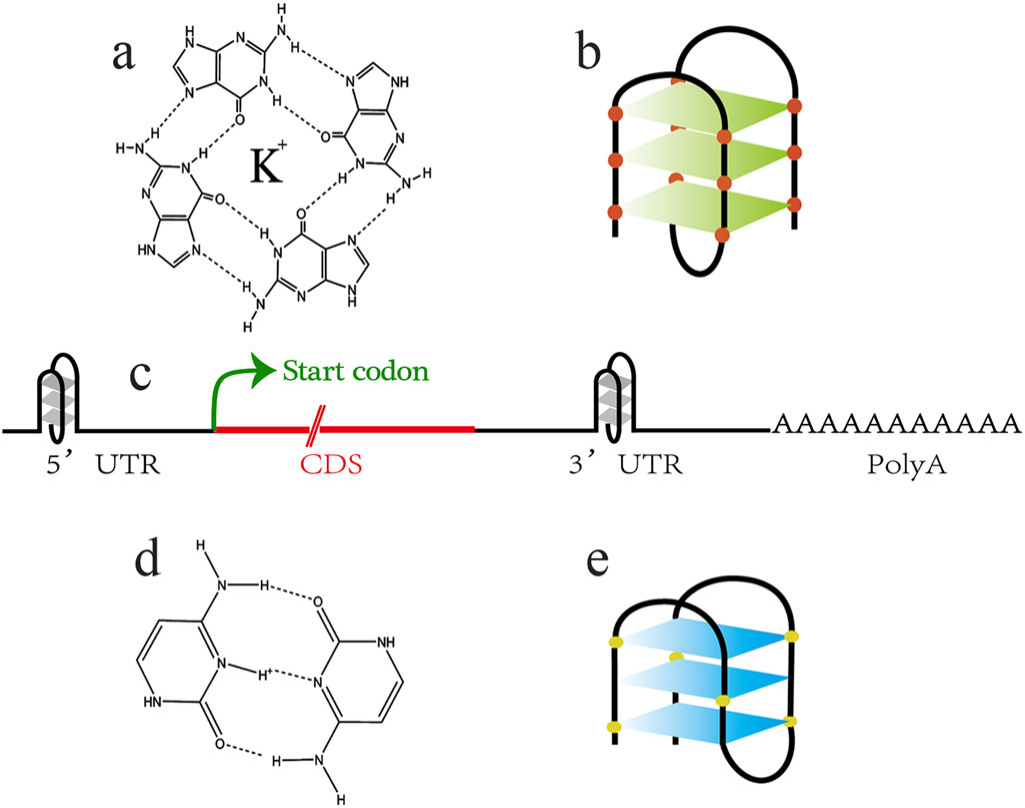
Schematic diagram of a G-Quadruplex (a, b) and an i-Motif structure (d, e). The secondary structure on the Untranscripted Region (UTR) of RNA may have effect on regulation of translation or post-transcription (c).

Certain guanine-rich nucleic acid sequences are apt to adopt four-stranded structures known as G-Quadruplexes (Fig. 10). Kumari discovered a conserved, intramolecular G-Quadruplex motif within the 5’-UTR of *NRAS*, which is a proto-oncogene in human, and have demonstrated that this RNA G-Quadruplex inhibits translation [55].

Unique putative G-Quadruplex forming motif has been defined in *Arabidopsis thaliana* by Mullen [56], following the pattern:

$$d{\left(G_{3+}L_{1-7}\right)}_{3+}G_{3+}$$

where d represents deoxynucleoside, G indicates guanine, L indicates any base, and + indicates “or more.” Considering that some sequences in EST may be negative strands, the complementary pattern d(G_3+_L_1–7_)_3+_G_3+_ was also used to predict the i-Motif. Details were shown in Methods. Using this pattern, twenty eight unigenes with G-Quadruplex were identified. Then the sequences were blasted against NCBI database to identify possible functions (S6 Table). The blast results showed that genes of phospholipid: diacylglycerol acyltransferase (PDAT), L-ascorbate peroxidase and some transcription factors had G-Quadruplex structure in 5’ UTR or 3’ UTR (bold and underlined in S1 Text). And i-Motif was enriched in the DNA binding factor genes in *S. sebiferum* (red and underlined in S1 Text).

L-ascorbate peroxidase has the function of hydrogen peroxide-detoxification and thus help the plants overcome the oxidative stress induced by abiotic or biotic stresses, for example, swAPX1 was highly induced in leaves by wounding, high temperature and bacterial pathogen in *Ipomoea batatas* [57]. According to Ding’s study, these stress-response RNAs were more plastic to change their structure in response to extreme environments, which may be a general mechanism of adaption for plants as sessile organism [13]. During flowering, these genes with hydrogen peroxide-detoxification activities were highly expressed to protect reproductive cells [58]. In *S. sebiferum*, the G-Quadruplex at the UTR of L-ascorbate peroxidase suggested the secondary structure may also play the important role in flowering process. Besides, the G-Quadruplex may also influence lipid biosynthesis and some transcription factors, which may be involved in flower development of *S. sebiferum*.

## Conclusion

Our research established a database of expressed sequence tags for *S. sebiferum* flower buds using Illumina HiSeq 2000 sequencing technology. MADS box genes and other genes involved in flower development were identified; the mechanism involved in drought inducing flowering of *S. sebiferum* was investigated primarily. All these results can enhance our understanding of the flowering regulation and provide more information for shortening vegetative growth of *S. sebiferum* through genetic modification.

Lipid biosynthesis, as primary metabolism, is conserved in seed, leaf and flower tissues. The main difference is the level of some relative gene expression. We applied transcriptome of flower buds to construct lipid biosynthesis pathway, which can provide information of some candidate genes for increasing oil content. Besides, as sessile organism, plant is always subject to various abiotic and biotic stress. Establishing the stress response pathway of *S. sebiferum* can improve its tolerance to stress conditions through transgenetic methods. The genes with G-Quadruplexes (GQS) were enriched in lipid biosynthesis and stress response pathway of *S. sebiferum*. The prediction of G-Quadruplexes provided another new aspect to understand the regulation of plant growth and development, including flower development.

## Methods

### RNA extraction, cDNA library construction and Illumina Hiseq 2000 Sequencing

All the flower buds of *S. sebiferum* were collected from test farm in Hefei, China. This farm belongs to Bioenergy Forestry Research Center of State Forestry Administration, an affiliation of Hefei Institutes of Physical Science, Chinese Academy of Sciences. All the plants in this farm were for research purpose. Inflorescences and flower buds at different stages (Fig. 1) were collected every two days in late April under natural condition (without designed drought stress treatment). Then these samples were immediately frozen in liquid nitrogen and stored at -80°C. RNA was extracted using RNAiso plus (TAKARA Co. Japan). Illumina sequencing was performed at Berry Genomic Inc. (Beijing, China) according to the manufacturer’s instructions (Illumina, San Diego, CA). Extracted RNA was qualified and quantified using a Nanodrop ND-1000 Spectrophotometer (Nanodrop Technologies, Wilmington, DE, USA) and Agilent 2100(Agilent Technologies Inc.). The 260/280 nm ratio of all the samples ranged from 1.9 to 2.1. The RIN value of each sample reached more than 8.0. All the RNA of different developmental stages was mixed with the equal amount. Then, cDNA library was constructed from the total RNA via reverse transcription, in which we applied oligoT primer to avoid rRNA contamination.

### Analysis of Illumina Hiseq 2000 sequencing results

Before the transcriptome assembly, stringent filtering process of raw sequencing data was carried out. Reads with more than 9-bp adaptor were removed before analysis. Then, the reads with more than 3% ambiguous bases represented as “N”, and with more than 50% with a quality score of Q<3.0 were also removed before assembly. *De novo* transcriptome assembly was performed by Trinity [59].

To understand the function of the unigenes, all the sequences were annotated with E-value cutoff of 1e-10 by blasting against public database: NCBI nucleotide sequence database (NT), NCBI nonredundant (NR), Swiss-Prot, Gene Ontology (GO), Kyoto Encyclopedia of Genes and Genomes (KEGG).

### MADS box gene prediction and Phylogeny tree construction

To build a HMMER model for searching translated *S. sebiferum* sequence, the Arabidopsis MADS box protein was collected from the Database of Arabidopsis Transcription Factors (DATF), then aligned by BioEdit. The multialigned sequences were trimmed in Jalview [60] to retain the MADS box domain. Then, the MADS box dataset was realigned by Bioedit and subsequently applied for HMMER search according to HMMER manual 3.0. Then we used *Arabidopsis thaliana* MADS box gene dataset [18] to classify *S. sebiferum* MADS box genes. A phylogenetic analysis was performed using all MADS box genes from the *Arabidopsis* and *S. sebiferum*. Maximum-likelihood tree was generated through MEGA with 500 bootstrap replications.

### Stress-induced flowering and semi-quantitative PCR analysis

During September 2013, Hefei suffered from severe drought. Interestingly, the two-year-old *S. sebiferum* in the test farm blossomed, which commonly flowered during late April to early May. We counted the number of flowering *S. sebiferum* in the test farm. Then, we collected leaves and flowers from the blossoming trees and leaves from trees without flowering. The samples were collected with three biological replicates.

To validate the drought stress inducing flowering, we carried out the experiment in a greenhouse, in which the conditions were maintained in a 16-h photoperiod at 25 ± 2°C with irradiance of 40 μmol m^-2^ s^-1^ provided by cool white fluorescent lamps. Fifty of one-year-old *S. sebiferum* were cultured in a separate garden pot. Each garden pot contained a mixture of peat and soil substrate with 100 cm^3^ volume. The fifty one-year-old *S. sebiferum* trees were divided equally into two groups with different treatments. One group was well watered as a control (approximately 100 mL water every two days) and the other was severe-drought treated (approximately 100 mL water every two weeks). The severe-drought treated seedlings bloomed nearly three months later. Then the plant leaves and flowers were collected separately and immediately frozen in liquid nitrogen when they were induced to flower.

Total RNAs were extracted using TRIzol (Takara Co. Japan) from the leave and floral organs. Each RNA sample was treated with RNase-Free DNase (Promega Co. USA) to remove any residual genomic DNA (gDNA). DNase treated RNA was subjected to reverse transcriptase reactions using oligo-dT primer and PrimeScript Reverse Transcriptase (Takara Co. Japan) according to manufacturer’s protocol. The gene-specific primers used in semi-quantitative RT-PCR were designed for the *GA1*, *CRY2*, *AP2*, *LFY*, *SEBIN*, *PRXII25514*, *PRXII39562*, *GRX50435*, *GRXII29453* genes (S2 Text). As a control, the cDNA sequence of the *ACTIN* gene was amplified by using the specific primers (S2 Text). The following thermo-cycling conditions were employed: initial denaturation at 94°C for 3 min; 30 cycles of 94°C for 30 s, 55°C for 30 s, and 72°C for 30s; final extension at 72°C for 10 min. The amplified products were separated on a 1.5% agarose gel, visualized and photographed.

### Prediction of G-Quadruplexes in EST

To search the potential G-Quadruplex-forming sequences throughout the entire EST, a regular expression pattern “G{3,}.{1,7}G{3,}.{1,7}G{3,}.{1,7}G{3,}” [61] was used in our own script program, which has been implemented by python 3.3.

## Supporting Information

S1 Table Summary of most abundant unigenes in the transcriptome of *S. sebiferum* flower bud. (XLSX)

S2 Table Summary of KEGG pathway. (XLS)

S3 Table Homologous genes in flowering pathway. (XLSX)

S4 Table FAD genes. (XLSX)

S5 Table DGAT and PGAT genes. (XLSX)

S6 Table G-Quadruplex sequence annotation. (XLSX)

S1 Text G-Quadruplex prediction. (DOCX)

S2 Text Primer sequences used in semi-quantitative RT-PCR. (DOCX)
