# Supplementary material for: Flower Bud Transcriptome Analysis of Sapium sebiferum (Linn.) Roxb. and Primary Investigation of Drought Induced Flowering: Pathway Construction and G-Quadruplex Prediction Based on Transcriptome
Source: PLoS One. 2015 Mar 4;10(3):e0118479. doi: 10.1371/journal.pone.0118479 (PMC4349590; doi:10.1371/journal.pone.0118479)
Supplement: S1 Text — (DOCX) [file pone.0118479.s007.docx]

>yang3.Unigene_13214|len=263|Ricinus communis Glycine-rich protein DC9.1

**GGGACCATGAGGGGGGTTAGGGCCAATAGGGGG**ATAAGGGCCAACAGGGGGTTTTTGGTCACCGCGGCCCTTCCCATTTCTGCCGTTATATCCACTGCGTCCATATTCGCCTATTCCCTTCCCATATCCGCCTTGCTCATATCCTCCATTTCCCTTCCCATATCCACCATTTCCATAGCCTTTCCCTTTACCATTGTAATTGTCAGCATCCACTTGTTCAGTCTGAGCAAGCACACGAGTTGAGACCTCAGAGGCAATGAGAA

>yang3.Unigene_24911|len=204|Ricinus communis phosphatidylinositol 3-kinase class

**GGGGGGGGGGGGGGGGGGG**AAGGAAGGTAGTACGGTTAGAAGAGATCAATTTTCCTAAGCTAGGCATTTACCAATTGGTCTTGTCGAATATCATCACCCTCCTTAAATATGATTTTGCAAGTACCTGTACCACCACTCTGTGTTTGGAAAGTCAGAAGCAATGAATGCAATGCACTTTTGAAAATTGAAGACTCGGATGGTACA

>yang3.Unigene_25704|len=412|Glycine max myosin heavy chain IB-like (LOC100777560)

**GGGCTGGGGTCCGGGACCTGGTGGG**TTCTTTGGAGGGTTTACTGATGGATTATGCAGCATGATATCTTCAGTGTTATCTTGCCTGTGCTGTTGTTGGTTGATACGAGATTGCTTTGGTGG

ACCAGGTGCTCCATATGGTTCTGGTCCTCCTGGACCTCGCCCACCATACTTCTGATCTACTTCTTGGCTAAAATGGCTTCGTTTGCTCTCTTTTTCTCTGTTTCATCCCCTATTTTTAGCTTTATATTTTGTTTTTCTTTTCTACTTTTACAGTTTTCTTATATTGCTATTTACCTCGTTGCATCAAAGAATTAATCCATTCTGTAATTCAATGAGCATATTCCCTTCTGCTATTAATTCATGTAATGAAGAACTACATAAAATATGAAATTTTCCAATTGTTTTTGCTAAG

>yang3.Unigene_53259|PREDICTED: Vitis vinifera DUF246 domain-containing protein At1g04910-like

**GGGAATGGGAATGGGAATGGG**TTTGAGAGAGGTAGGGTTTAGATTCATTTTCCAATACGTGCCGAGTGGTAAAAGCAAATATAAAAAGTGAAAAGGCAAAGAGAAGGATAATGAAAAGAGTAATAGCAGAAGGAGAAGGTTTTCTTAAGAGAAATGGAGCTGCTGATGAGCTTTTGATGTGAGAATACCCTCTGCTGTTCACCATCTCCAGAGCTACCCCTCACTCACGACTTCTTCTCTGCTAAAATTTCACTCGCTGGTTTAATTGTGTACCGTACCGTCGTTTCAACTGAAACTGGGAAAT

>yang3.Unigene_128211|len=2851| Ricinus communis phospholipid:diacylglycerol acyltransferase 2 (PDAT2)

**GGGGGGTTCGCGGGGGTGGGTGGGTGTTGGGTGGGACGGTGG**AGAAGCAGCAGAAGTCAAATTCCATACACTTCAGTAACATGGCAAGCAACATTACTGATCTTCTCCAGAAATTTTCCTGCAAAGTACTTGTTTCTGTCATGGTTACAGCAGCAATGGGATTAAGAACGAGACTTCAAAAAATCCTGCGACTTCCTTTGTAGTTAGTTCTGTCTTTGAGCCTCAAAGCCAATCATCTCCCTTTCTTTATGGTAAAATTGTTGATAAGTCTTGATGTAATTACAGACGAAGATTTATTTTCTCGGACATTCTTAATAGATCAGAATGAATCCTGTCACCACCAATTTCAGGACCACTAGCACCTGCAGCAACTCTTAGTACATCTTCAATAAAAGCAACATTTCCCATAATGTCAACATGGGAACCACTCCCTGTACCTCTCCCTTCAAGCAGGCTAGCTGGTGGTTTGTGCTCATACTCCCTCACATATGTAGGACTGCCCGACGGGTTGAACCGGGTTTTCCCTCTCCAACCTTTAGCACACATGTAGCCTGAACTAATCACAGGCACACTTTCATCACCATCAGCAAAGAATACTCCCCTTTTTAAGTTACTGTCCTCTTCTCCATCAACTGAGGCATCGATTCGGAATGGAAGAATCTTGCATCTATCAGTTGCTGATAACTTATAGATGTATGATCTTTCTGTTGGCAGTCCAACCCCATATAAGCAGTATATCTCCAGTTCAGGGGCATTAGGCAACCTGACAGAAGAAATTCAACTCTCATAAGTTCAAGTGGTGTTTGCCTGATTTCACGACTAACAAAAAATATATGCATTGATGCTCTTACCTTGTCTCTAATGAGTTAGACCAATATTTGTAATGTTCATATTTTGGATCATCAAGATCGTCGGCTATCCCATTTGAGAATTGAGCTTCAAGACGTTGCATCATTTTCGGGGCAACAAATCGAAGCAAATCAAAAGTAGAAGCAACTGTAAAAGCTTTATTTTCCACAAATTTTCGAATATTTTCCCTGTTTATTTCATCATATTCGGTCCAAACCTCTTCACATGATGATCTTGAATTCTCGTTGGTAGCGAATAATTCCTTTGAATCAAAAGAAGGAAGCTGTGAAGATGGCAATTCTGATGCTGCCTTGCTAAAAGAAACTATCCTTCCATACTTAACTGAATCGTTTACTGAAGACACTTCAGGAGACCAATCTAAGTTACCCCAAATAGTCTCTCCTCCTTTAGGCATCATTGAAAATAGTGAATCCCACGTTCGAAGCACCCGCAAGCAATTCTCCATGGTATGAAACCAGGGAGTTTCAGAATCCCAAATGCCAGGAAACCTAGCTCTGAAGTCTGCAGCGTATTTGTTTTCTCCAGAGAGTAGATGAGTTATTGCCTTTGGAGTACCCAGAAATGTCGGACCGATGTTCATGATTGATTTTATGTACTTGTTGCACCACCCTGGACTGCCACCGCCGCCCATTGGCGAAGGTGCTTCAACCCATTTGAGGAAGTACAGAAAATACTGAACCCCCATGGAATGAGGAACCACCACCACTTTCTTATTGCCATTTGCTCCATACATGAACTCTATTTTACTCTTCAATTTACTAAGTGAATGGTCTCGAATCTCTGTATTCTGAAAAGAGATCCTCCAATCATAGGTTGCCATATATAGATTATTCCCTTCGTAGCCAATTTTGGCCAAATTCTCGATTAGAACAGCCCAAACAAAAAAACCCAGGAGCAAAATAGTCAGCTGCAGCCAGCCCTGGGATCGGACGAACTCGGATGCCTGGCGGGTCAAGACCAGTCTCATTGTCCAAAGACATGTGATCCAACCAACATGATGGCCTTCGGAACATTCCACTGAAGGTACCACCCCAAAGCCTCTTTCTGAAAAGACCATGAGCACAAGGTCTGCCTTCCCATAGCTCAAGGCCACCAGTGATAGTGCCTGGCACCAAAATCACTGGATGTTTAGCTGTTAACCCTTCTCTTTTTAATCTGTCTCCTACCGATTCAGGCAATGTAGCCTGCAAACTGTGGTACAAGAACAGAATCAGCCACCAAGTGGTCCATATATACCCAATCATCCGGCAACCGCAGTCTATGCAACTCCATGCTTTTGGCTTCCTTTTTCTCCTTTTATTGTTAATTTCCAACTCGTTTTTGGTATCAGGGTTGACAATTTCTTCTTTCTTGTCATATTTTTGGGAATCAAAAGACTGTTTGATAGTAGAATGGACAATTTCTTCTTTCTTGCCACATTTTTTGGATTCAAAAGATTCAGAATTTTCTGGCTCTACACAGCACAGTTTTCGAAACCGAAGAATTGAAGCCATGATATATTAAGGAAAAAAAAACTCAAATTAAATCACCAACAAGAACAACAGATTTCAGGTAATTAATGTGAGAAACAAGAAAGCAAGAAAAACCCAGAAAGGGAAAATAATAATATATTAGAGTATGGTAGATGAAGAGGTAGCAATGGTGGTGAGTTGTGGTGAAAACAAGGTTATGGGCTATAAGTATCTGTTACAGAAAAAGCAGAAAAACACGTGGGGTGAAGAAGAGAGATGAGATGGGCGTAAACCATTTCTCCTTCTCACGGTGTCCACATCCATAATTCCATTTGCTTTATTAGCATGGAACGATTCAAGAGGATTTGTTTATGCTTACTACATGCTTCGTACAATTTCAGACATTCTAAGTCAAGGAAGCATATTCTGCCTTTCTGTTGCATATGCAGTAAGAATTAGTGTCTACTGTTGAGAAACCATAGACATAAGAATGCAGAGGCATCAATTTTTTCCCCTGCATTTGTTAGGCA

>yang3.Unigene_90768|Ricinus communis transcription factor

**GGGGGGGGGGGGGGGGGGGG**AAAGCTTGTCTAAAAGAAAGCAAATCTTCTAATAATACCGCATTAGATACGTGCATGGCATATATATGGATGGATTAGGCTACAAGGTTAACATTAATGGACATAAATTACAAGATTCTATATATATATATAGATGCATTTTGAGTGGATGATCTGAATTAAGATCTAGAGACCAAGATTCTTCAGGGAGCTTGATAGGAGCATCTAACATCATGTGAAAGCCGACCCTGTTGAAAGGAACTGAAGTGAACATGTCATGGGCAGTGATGTCTGAATCAGTAGGAGCAGCTGCTGAGATCCCTATTCCATTACAATCCATATCAAATGACAAGGGTGGGTATTGGTTTGGGTTTGGATGAATCATATCCACCCAGCTCAAGCTGGCATCAGGTAGTTGAAAATTGGAAAAGTCTGCCTCTGTCTTGCAATGTTTTTGAATAGAGGATTGTTCCTTCAAGGGGAAATCCTGATGTGAGAGAAGGGACTTGAAGAGCATCGATGGTGACGGGTTGCTGTCGGTGTTTGGATCTATATTTTTTTTTGTTTCTGCTGCGTTGATTGCCATGGAAGCGGGTGAAGAAAAACAGGGTTGCAACCCATTTATTGAGAAGAGATTATGAGATTGGGAGACTAAAGGGCTTAGAACTGAGCTTTTAAGATCATTTTCTTGGCGAGGAATTAGAAAAGGATTCTGCATTGCCTCCGATGATGTCTGTGATTGAGATTGACATTCTAGTAATGCAGAAGGGGCTGCTTCAAGCAATGAAGGCAAGTAAGTATAATTGTGGGGTGAAATAGCTTCTAGGAGGAAGCCTTGCCCTTGCAGAAATCCTTTTTTCTTCTCCCCAGTTTTGTGAAACATCCTGCAAATCATCCATTCCGCCTTACACGATCGGCAGTTGGAGAAATCACCGTCGAGGCGGTACTCGTGCATGACCGACCCACTTAGTTTTCTCACCTCTAGGAGCTCTGCCCTTATAGTAAACAAGGATCTTTTTCATGCCAAGTAAACACCACTTGAAGCACTGTATACTTCCCTATCTTTCCCTGTAGCTTTCCAGTACCCAGCTCCTGTTGCTCTATTTGTTCTCAGCCCTGTTGGGTACTTCCTGTCCCTCAGGCTGAAGAAGTACCACTCTCTTTCCCCCATTTTAGCCACATTTTTTCACAACAATACCCATTAATTACTCACACATTAATTTACTCATTTTGGATTCCTCACAAGTTAATTAATCATACCCAAAAGTTGTGATATATTTGTAGCTTAATTAAAGAATGAATAAAATCTAAGCATGTAAAAATCTGCAAGTCTTATTAATTAGATATTAATCAGTAAAAGAGAGAGAGATAGTATGCATTGCAAAAATCCAAAAATCACCACCAGTTGTAAGAAACAATTACAAAGATGACAACTTTCTGCTTTGCATAGGTTTAAGGCGTCTTGTGGTGTGGGTTGGGACTTGGGACTCTGTATGCAGAACCTTTAAGGCATAGCATCGAGCAACATGCCAGAAAGTGTCCAAGATTGATGGAAATGCAACTAAAGTGGAAGGGAAGATTCTTATTTTGTGATCAAATGTGGAGTGACAAGAATTTCAAATAGCATTTGATAATCCAAAAAGAAGAGAAAGAAAAAGTTACCAGAAAGCTCCCAAGGCTCACATCTGTTGAGGTCAACCTCAGCGATTTCGACTCCACAAAAGCTGCCATTGAAGACCTTGGAAGCAAGATAAAATGTTATAAGCTCTTCATCAGTTGGG

>yang3.Unigene_90774len=1664|Ricinus communis transcription factor

**GGGGGGGGGGGGGGGGGGGG**AAAGCTTGTCTAAAAGAAAGCAAATCTTCTAATAATACCGCATTAGATACGTGCATGGCATATATATGGATGGATTAGGCTACAAGGTTAACATTAATGGACATAAATTACAAGATTCTATATATATATATAGATGCATTTTGAGTGGATGATCTGAATTAAGATCTAGAGACCAAGATTCTTCAGGGAGCTTGATAGGAGCATCTAACATCATGTGAAAGCCGACCCTGTTGAAAGGAACTGAAGTGAACATGTCATGGGCAGTGATGTCTGAATCAGTAGGAGCAGCTGCTGAGATCCCTATTCCATTACAATCCATATCAAATGACAAGGGTGGGTATTGGTATGGGTTTGGATGAATCTTATCCATGGCATCAGTTAGGTTAAAATTGGAAAAGTCTGTCTCTGTCTTGCACTGTTTTTGAATAGAGGATGGTTCCTTCAAGGTGAAATCTTGACGCGAGAGCAGGGACTTGAAGAGCATCGATGGTGACGGGTTGCTGTCGGTGTTTGGGTCTATGTTTTTTTTTGTTTCTGCTGCGTTGATTGTTATGGAAGTGGGTGAAGAAAAACAGGCTTGCAACCCATTTATTGAGAGGGGATGATGGGATTGGGAGACTAAAGGGCTTAGCACTAAGCTTTTAAGATCATTTTCTTGGCGAGGAATCACAAAAGGATTCTGCATTGCCTCCGATGATGTCTGTGATTGAGATTGACATTTTAGTAATGCAGGAGCTGCTTCAAGCAATGAAGGCAAGGAAGTAGAAATGTGGGGTGAAATAGCTTCTAGGAGGAAGCCTTGCCCTTGCAGAAATCCATTTTTCTTCTCCCCAGTTTTGTGAAACATCCTGCAAATCATCCATTCCGCCTTACACGATCGGCAGTTGGAGAAATCACCGTCGAGGCGGTACTCGTGCATGACCGACCCACTTAGTTTTCTCACCTCTAGGAGCTCTGCCCTTATAGTAAACAAGGATCTTTTTCATGCCAAGTAAACACCACTTGAAGCACTGTATACTTCCCTATCTTTCCCTGTAGCTTTCCAGTACCCAGCTCCTGTTGCTCTATTTGTTCTCAGCCCTGTTGGGTACTTCCTGTCCCTCAGGCTGAAGAAGTACCACTCTCTTTCCCCCATTTTAGCCACATTTTTTCACAACAATACCCATTAATTACTCACACATTAATTTACTCATTTTGGATTCCTCACAAGTTAATTAATCATACCCAAAAGTTGTGATATATTTGTAGCTTAATTAAAGAATGAATAAAATCTAAGCATGTAAAAATCTGCAAGTCTTATTAATTAGATATTAATCAGTAAAAGAGAGAGAGATAGTATGCATTGCAAAAATCCAAAAATCACCACCAGTTGTAAGAAACAATTACAAAGATGACAACTTTCTGCTTTGCATAGGTTTAAGGCGTCTTGTGGTGTGGGTTGGGACTTGGGACTCTGTATGCAGAACCTTTAAGGCATAGCATCGAGCAACATGCCAGAAAGTGTCCAAGATTGATGGAAATGCAACAGGAAGCTCCCAAGGCTCACATCTGTTGAGGTCAACCTCAGCGATTTCGACTCCACAAAAGCTGCCATTGAAGACCTTGGAAGCAAGATAAAATGTTATAAGCTCTTCATCAGTTGGG

>yang3.Unigene_95967|len=1691| Ricinus communis L-ascorbate peroxidase, cytosolic

**GGGGGGGGGGGGGGGGGGGG**AGAGTATTGATAAGCATCATATATGTTATGTTAGGAAGAAGAGAGGGCTCTTCCAGAGTGTGGGTCTGTCGCTGGTTAATTGGGTGAGGACTGATTCTAGATATTTCTGACATGGGTTGTTGAAGAGTAGTATAGTGAATATGTTTAGGAAGTTTGTTACATAAGGAAATGTATTAAGGGTATATGAGAGAGGTGGGAGATGAGTAATAGCATTCTGACTTTAAGTAAAGGTTTTAGTAACCATGGAGAGTCACAAGCCTCTCGAATGCTTGTAATTACCTATTTTCCAGTTCAATTCATAATTCTCTTTTTGATTCCTTGTGTTCTCGTTGGTTCTTATCAATTGGCATCAGAGCAAGTGATCCGTGGAGAAGATGAATTTTGGGCAAAGATTGGAGACTAGAGTTGACATCTTTGCAAAAGTGATGCGTTCTCTGAAGAACACGGTGCACAAAATCTCAATGAAGCTAGACAAGCTCATGGAACTGTGTAAAGACTCATCCAGGGTGGCGCCACCGCCCGGGGAAAGGCAAGATAGGGAGGAGGAATCATCAGAGCTAGAGTAGAGATCGACCATGCCTAAACTCCCTCCGATGCACACAATAGGTGATGGAATCCTTGTTTTAGGCCTAAGATTGGATCGACAACTATTGTCTCCAAACCCTAAATCGAAGGAGCTTCAAAATTCCCAAAATCTTAAAATGGATCATGAGATCCATGCCCAACATAAGAGTGCAGGTTCCCTCTTCAAATCACACACCCATCGACGGAGTGCCTTGCTCGATTCCGTTCTCCCAACTTCAATGAGCGCAATGGTTGTATTAAGGGAGTGGTCAACGTTCTCATCCATGATCCTGGTGGCAGATCTCTGCTGGTCCGTGTCTCTTTCCGTCGCGCATTCCAGTACACAAGCATCTGAAGAAATTATACGAAGCTGCTGAAGAGATTTATTTTGGTCAGATGGTGACCAACGACAGTAAGTTACTTATTTACGAGATCAAAAAGACAGTTGAGAACTTGCCATTCATGGATTGGCTCAAGAATAGGCGTCATTATATTTTGTTTATGGTTGATGCTATTGATGAGTACGTTGTGCAGAAACTGAAGGAATATGATAAAAAGAAGCTTGTTTCGGCTACAAAGGATTGAAACTTGATGATGGGACTGAAGTAGTTGACAGATATACAATCCAAGAAAAAGAAGTTCAGAGGTTTAATTGACGAGAAGGATTGTGTTCCTATCATGCTTTGTCTCGCATGGCACTCTGTCGTCGATGATTACAATGTGAAGACCAAGATCAATTGCCCATTTAGGACCATCACGCATGCAGTAGAGTTGGTCCAGGGGGCCAATAATGGGCTTGAGTTTGCTAAAGACTACTTGAACCCAAGAAGAAGCAGTTCTCCATTCTCAACAACTCTGACTTCTATTAGCGGGCCAGTGGTGTTGCCCGAATTGGGTTCTATCTCATCTCTCTTCCTCTTTTAGATCGGAATTTCTCAAACCTCTTTCTGCCGCCATTCCCCGTCGTCCGATCTCCTCCACCACCAATCCATTCACCATCGAGACCTCCGTTTCCTTTCTTATTTCTAGACATGCAGCCATAATACCACCATTTTCCAAGACATTCGCATCCCAAAAGCAACATGGATGCTCTATACTCTGAATGCA

>yang3.Unigene_95969|len=1604|Ricinus communis L-ascorbate peroxidase, cytosolic

**GGGGGGGGGGGGGGGGGGGG**AGAGTATTGATAAGCATCATATATGTTATGTTAGGAAGAAGAGAGGGCTCTTCCAGAGTGTGGGTCTGTCGCTGGTTAATTGGGTGAGGACTGATTCTAGATATTTCTGACATGGGTTGTTGAAGAGTAGTATAGTGAATATGTTTAGGAAGTTTGTTACATAAGGAAATGTATTAAGGGTATATGAGAGAGGTGGGAGATGAGTAATAGCATTCTGACTTTAAGTAAAGGTTTTAGTAACCATGGAGAGTCACAAGCCTCTCGAATGCTTGTAATTACCTATTTTCCAGTTCAATTCATAATTCTCTTTTTGATTCCTTGTGTTCTCGTTGGTTCTTATCAATTGGCATCAGAGCAAGTGATCCGTGGAGAAGATGAATTTTGGGCAAAGATTGGAGACTAGAGTTGACATCTTTGCAAAAGTGATGCGTTCTCTGAAGAACACGGTGCACAAAATCTCAATGAAGCTAGACAAGCTCATGGAACTGTGTAAAGACTCATCCAGGGTGGCGCCACCGCCCGGGGAAAGGCAAGATAGGGAGGAGGAATCATCAGAGCTAGAGTAGAGATCGACCATGCCTAAACTCCCTCCGATGCACACAATAGGTGATGGAATCCTTGTTTTAGGCCTAAGATTGGATCGACAACTATTGTCTCCAAACCCTAAATCGAAGGAGCTTCAAAATTCCCAAAATCTTAAAATGGATCATGAGATCCATGCCCAACATAAGAGTGCAGGTTCCCTCTTCAAATCACACACCCATCGACGGAGTGCCTTGCTCGATTCCGTTCTCCCAACTTCAATGAGCGCAATGGTTGTATTAAGGGAGTGGTCAACGTTCTCATCCATGATCCTGGTGGCAGATCTCTGCTGGTCCGTGTCTCTTTCCGTCGCGCATTCCAGTACACAAGCATCTGAAGAAATTATACGAAGCATTCATGGATTGGCTCAAGAATAGGCGTCATTATATTTTGTTTATGGTTGATGCTATTGATGAGTACGTTGTGCAGAAACTGAAGGAATATGATAAAAAGAAGCTTGTTTCGGCTACAAAGGATTGAAACTTGATGATGGGACTGAAGTAGTTGACAGATATACAATCCAAGAAAAAGAAGTTCAGAGGTTTAATTGACGAGAAGGATTGTGTTCCTATCATGCTTTGTCTCGCATGGCACTCTGTCGTCGATGATTACAATGTGAAGACCAAGATCAATTGCCCATTTAGGACCATCACGCATGCAGTAGAGTTGGTCCAGGGGGCCAATAATGGGCTTGAGTTTGCTAAAGACTACTTGAACCCAAGAAGAAGCAGTTCTCCATTCTCAACAACTCTGACTTCTATTAGCGGGCCAGTGGTGTTGCCCGAATTGGGTTCTATCTCATCTCTCTTCCTCTTTTAGATCGGAATTTCTCAAACCTCTTTCTGCCGCCATTCCCCGTCGTCCGATCTCCTCCACCACCAATCCATTCACCATCGAGACCTCCGTTTCCTTTCTTATTTCTAGACATGCAGCCATAATACCACCATTTTCCAAGACATTCGCATCCCAAAAGCAACATGGATGCTCTATACTCTGAATGCA

>yang3.Unigene_99739|len=1242| Ricinus communis DNA-directed RNA polymerase

**GGGGGGGGGGGGGGGGGGG**TGGGGCCAAAGGGAGATGAAGTATATATGAATAATTTTCCAAACAAAGTCGTTAATGTATGATTTCAGATGATATTATGCTAAGTTATGTTGTTGCAGCTTGCACTGATTAATGGTTAGCATATTGTTCTTTTGCCAATCAAAACCCTGGTTTCTATCTACTAGCCTTGACTAATATTGTATTTGAAATTTTTGATTGGTTGAAAATAATACCGAGCAATTTTCATTTTTTACTGTTGTTTGATAGTCTACAGCTTCAGTTAGTGACTGCCCTATTAGCCATTCAAGTCAGCTCACAAATCCATTTCTTGGTTTGCCGCTTGAATTTGGTAAATGTGAGTCTTGTGGAACATCAGAACCTGGAAAATGTGAAGGTCATTTTGGATACATTGAACTCCCAATCCCAATATTTCATCCTAGCCATATTAGTGAACTGAAGAGAATGCTTAGTTTATTGTGTCTGAAGTGCTTAAAACTTAAAAAGTTTTTGGCTAAAAGCAATAGTGTGGCTCAGCAATTGATGTCATGTTGTGAGGAGGTTTCACATGTTTCAATTAAAGAGATTAAAACATCAGATGGTGCCTATTTTTTGCAATTAAAGCTACCATCTAAAAGGAGTTTGAAAGAAAATTTTTGGAATTTCTTGGAAAGATATGGTTTTCGCTATGGTGACAACATTACTCGGACGTTGCTGCCTTGTGAGGTGATGGAAATGTTAAAGAGAATTCCAATGGATACTAGAAAGAAGCTCGCCAGAAAAGGTTATTTTCCTCAAGATGGATATATCATAAAATATTTACCTGTTCCTCCAAACTGTTTGTCTGTGCCGGAAATTTCAGATGGTGTTAGTGTCATGTCCTCGGATCTTTCTATATCTATGCTCAAGAAAGTTCTTAAGCACGTTGAGATAATCAAAAGCTCAAGGTCTGGTCCACCAAATTTTGAGTCCCATGAAGTTGAAGCCATTGATTTACAGTCAGCAGTGGATCAGTATCTTCAAGTTAGGGGCACTGCAAAGGCCTCACGTGATGTAGGAGATCGATTTGGAATCCACAAGGAGTCCAATGAATCCTCAACAAAAGCGTGGCTTGAGAAAATGAGAACATTGTTCATAAGAAAGGGCTCTGGATTCTCTTCGCGAAGTGTGATTACAGGTGATGCATATAAGCAAGTCAATGAAATTGGGATACCATTTGAAATTGCACAAAGAATCACCTTTGAGGA

>yang3.Unigene_123966|len=2919|Ricinus communis protein phosphatase 2c

**GGGGGGGGGGGGGGGGGGG**AAGGATGATAAAAGAAACAGAGGAAAGGCCCCTTCTTTTGTACTTTTAATTTTCTAAGGCGCGATCCGATCCTATCAATCCCGTCCACATATCTCCCTTGTATATATATATATATATATTTAGTCTTCAGCCTTTGGGATATCTACTGAACTATTCACGGCTACCTAGGCTAGCATTAATCTTGGCTAATATCCTTCGGAGTTGCCCGTTATCTATGCATATAGCACCCAAAGTATATGTCCTATGATCCACGAAAAAATGGGTCATGTTTTCGATTCTCTATCATGCTTCCAGCAGGCTCAACTTGAAAATTGAAACTGAATTCATCATTCTCACTTTTACAGAGCTAGTTCACGGTTGAGATACCACAATGTACCGAGATTTCAAAGATATTTTCCTGATGACTTCCAGATTCTTCCTTCAAGAGATATCACCATAACAGTAACATCATCATGGTACTTCCGCCTATCTCCTTGTGGGATGTCTAGCAATTCATGGAAATCCATACCAGCTTTCTTGGCTGCACGAGAAAGAAGCTCTTCTATCAGGTGCTGTGCTGGGTCACCATCTGGAAACTTCTCCATGAAACTCTCAATATGAGAAACCACTTCCTGATTGCTCAGATACTGATATAATCCATCAGATGATAGGACTAGAAATTGATCTCTTGGACACAATTGATGGTGATGAAGAGAAGGTATGCACGACACATATGGTGCACTGCCAATAAACTCATTCCGAAACATCTCCAGTAATGCATCATTAAATTTGGACTGTTTTAGAAATCCTGCACCAAATGCTCTGGTAACCTTCAGACGTCCTTTTACTCTATCATTGACAATACATTGACTATCATCTGGGTGTTCATTCTTGATTCTTAAGACTTCCTCTTCAATGCTTGTGCTGTGATCAGTGGACAGCTGCAATGCTGTCAACCTCATCGCCTGTGCAGGACCCCCAGCTAACCCCTCCATGTTCATCGTCCGTGTAGGACCCTCATTTAACCCCTCCATGTTCAACCCATTATCACCCACACTAGAGCAAACCTCTTGTGGCTCATACTGCGCAACAATTGCTCTGCTATCACCTACATTCATCACATACACGTCCTCATCTCTCATCAACGCCACCAATAAACATGAGCCCATCAATGCTAACTCCGGATTTGTATCAAGCACCTTATCAGTCATATCCAAATATGCTAATTCTGTCACCTCTAGCGCCCTTGATAATGCCCTCAAAACCAATTCATGGTCTGCAGGTCCCTCCTTCCGCTTCCTTTGCAAAGCCATCTCCTCCACTCTACTGCTCTCCACCTTCTCCTCCTTGCCCTCCAATCCAAATTTCCATGGAAACAAGTTTTTCTCCCTGTGTTTAGACAATCCTTGTTTCAGCTTTGACAGCAACAACCACCTCTTACTGACAGCAGAACCTGCATTGCCCACACTAATTGCATCATCAACTGAAAATGCAAATCTATCAGACCCCGAAAGATCAAGCCCGTCTTCTGCATTTTCTTCTGCAAGAAATTCCCATAATCGCCTTCTCTGAGTAATCTCCGTCCCCACTGACTGAAACGTAACCCTCTTTGTCCTTTCATGAGGCGCCAAGCTCGACCCAATTTCCCCGATTGAAGCCCCCTCGTCCTTTTCAACACCAATTTCATTTGCTGGATTAGGATTAGCTTTAGCTGCATTTTCTTGATCTTTCGACGTTATATTCGATTCCCTCCCACCAATTGAACTCTCCCCAACACTTCTACCGCTCTTTCCTAGTGATAGGTTAGTTCTACTTTCACTTTCCTCTACAATTGTTGATGTTACACTACACACTTCGGCCTCTTCAATGTCCCAAAACAGTCCTTGGAGTTCATTATACACAGCCCTATAGAGATTCCCCATCAAGAACTCCGGTGCATCAGGGCCGTTAAACCCATCATAAATCCCAACAAACAACCATCCCTGCTCCTCCGATACAACCACATGGACCCGATCCTCTCCTGCTTTCCCCAAGGCCCACTGTACAGTACTCTCATCTTTGCTCTCCTTCTCCTCCACGGTACCCGAATTCTCTTTCCGGTTCACGAAATTCAGTACCGGGACCACCCACGGCCTTTTCTTCTCCGATAAGTTCCGATAAATCGCTTTCTTAATGCCAGAAATGCCCTTCCTCCGCTTCTTTTTCACATAAATGCCACCGAGCGGCGCCGAGAAGTGCACCCGACCGCTATTGTCGGTCCCCGAGTTGGCGTCAAGCGGACCGGATAGTGCACCACGTTCAATGGGACCCGACATAAAGAACCCCTCTCCACCACGTGGCACTGGTTGCAAGCGTAAGGCATTGAACGACGCCGTGCTCTCAAACCCATCGACGTTGACAATACTGCTCTTTACTCCACCACCGGTAACACCAGCAGCAAAGCCAGAGCTGTCAATGGCATCATCGTAAATATTATCAAGTTGAAGTACAGTCCTGGGCGTTGAAGTGTTAGCACTAACCGAAGCACCGGAAATGGACTTAAACCCGGTCTCGGGAAATCCGGGCCGGGTCTCAGAAACGGCCCCGACCCGAGAAGGAGATAAGCGGAGAGAGTTAGAGGGAGAAACAAAGCGATCGGAAGGAGTAGGAGAGACGAAGTGGTTAGAAGATCGGACGTAGCGGAAGGAATGACCTAGGGTTTCATCCAATGGGTCAGCGGCCGTGAAGATCAAGTCCGGCTGGTCATGTTGAGTCCGGTTAGTCGCCGGTTTGAAGCAAGGGAAGAGGGAGGAGAGTCCACTTCCCATCACATTTGATTCGCCACCTTCCGTTTGGACTTCAAGAAATCAAAAGAAAAAAAAAAATTTCCTTTTCTTTTACCTTTCTTTTGAAATTAAGTTCCGGCAAAGTTTTGAAGAAAA

>yang3.Unigene_123968|len=2880|Populus trichocarpa phosphatase 2C family protein

**GGGGGGGGGGGGGGGGGGG**AAGGATGATAAAAGAAACAGAGGAAAGGCCCCTTCTTTTGTACTTTTAATTTTCTAAGGCGCGATCCGATCCTATCAATCCCGTCCACATATCTCCCTTGTATATATATATATATATATTTAGTCTTCAGCCTTTGGGATATCTACTGAACTATTCACGGCTACCTAGGCTAGCATTAATCTTGGCTAATATCCTTCGGAGTTGCCCGTTATCTATGCATATAGCACCCAAAGTATATGTCCTATGATCCACGAAAAAATGGGTCATGTTTTCGATTCTCTATCATGCTTCCAGCAGGCTCAACTTGAAAATTGAAACTGAATTCATCATTCTCACTTTTACAGAGCTAGTTCACGGTTGAGATACCACAATGTACCGAGATTTCAAAGATATTTTCCTGATGACTTCCAGATTCTTCCTTCAAGAGATATCACCATAACAGTAACATCATCATGGTACTTCCGCCTATCTCCTTGTGGGATGTCTAGCAATTCATGGAAATCCATACCAGCTTTCTTGGCTGCACGAGAAAGAAGCTCTTCTATCAGGTGCTGTGCTGGGTCACCATCTGGAAACTTCTCCATGAAACTCTCAATATGAGAAACCACTTCCTGATTGCTCAGATACTGATATAATCCATCAGATGATAGGACTAGAAATTGATCTCTTGGACACAATTGATGGTGATGAAGAGAAGGTATGCACGACACATATGGTGCACTGCCAATAAACTCATTCCGAAACATCTCCAGTAATGCATCATTAAATTTGGACTGTTTTAGAAATCCTGCACCAAATGCTCTGGTAACCTTCAGACGTCCTTTTACTCTATCATTGACAATACATTGACTATCATCTGGGTGTTCATTCTTGATTCTTAAGACTTCCTCTTCAATGCTTGTGCTGTGATCAGTGGACAGCTGCAATGCAGTCAACCTCATCGCCTGTGTAGGACCCTCAGCTAACCCCTCCATGCTCAACGCATTATCACCCACACTAGAGCCAACCTCTTGTGGTTCATACTGTGCAACAATTGCTCTGCTATCACCTACATTCATCACATACACGTCCTCATCTCTCATCAACGCCACCAATAAACATGAGCCCATCAATGCTAACTCCGGATTTGTATCAAGCACCTTATCAGTCATATCCAAATATGCTAATTCTGTCACCTCTAGCGCCCTTGATAATGCCCTCAAAACCAATTCATGGTCTACAGGTCCCTCCTTCCGCTTCCTCTTCAAAGCACTCTCCTCCACTCTACTGCTCTCCACCTTCTCCTCCTTGCCCTCCAATCCAAATTTCCATGGAAACAAGTTTTTCTCCCTGTGTTTAGACAATCCTTGTTTCAGCTTTGACAGCAACAACCACCTCTTACTGACAGCAGAACCTGCATTGCCCACACTAATTGCATCATCAACTGAAAATGCAAATCTATCAGACCCCGAAAGATCAAGCCCGTCTTCTGCATTTTCTTCTGCAAGAAATTCCCATAATCGCCTTCTCTGAGTAATCTCCGTCCCCACTGACTGAAACGTAACCCTCTTTGTCCTTTCATGAGGCGCCAAGCTCGACCCAATTTCCCCGATTGAAGCCCCCTCGTCCTTTTCAACACCAATTTCATTTGCTGGATTAGGATTAGCTTTAGCTGCATTTTCTTGATCTTTCGACGTTATATTCGATTCCCTCCCACCAATTGAACTCTCCCCAACACTTCTACCGCTCTTTCCTAGTGATAGGTTAGTTCTACTTTCACTTTCCTCTACAATTGTTGATGTTACACTACACACTTCGGCCTCTTCAATGTCCCAAAACAGTCCTTGGAGTTCATTATACACAGCCCTATAGAGATTCCCCATCAAGAACTCCGGTGCATCAGGGCCGTTAAACCCATCATAAATCCCAACAAACAACCATCCCTGCTCCTCCGATACAACCACATGGACCCGATCCTCTCCTGCTTTCCCCAAGGCCCACTGTACAGTACTCTCATCTTTGCTCTCCTTCTCCTCCACGGTACCCGAATTCTCTTTCCGGTTCACGAAATTCAGTACCGGGACCACCCACGGCCTTTTCTTCTCCGATAAGTTCCGATAAATCGCTTTCTTAATGCCAGAAATGCCCTTCCTCCGCTTCTTTTTCACATAAATGCCACCGAGCGGCGCCGAGAAGTGCACCCGACCGCTATTGTCGGTCCCCGAGTTGGCGTCAAGCGGACCGGATAGTGCACCACGTTCAATGGGACCCGACATAAAGAACCCCTCTCCACCACGTGGCACTGGTTGCAAGCGTAAGGCATTGAACGACGCCGTGCTCTCAAACCCATCGACGTTGACAATACTGCTCTTTACTCCACCACCGGTAACACCAGCAGCAAAGCCAGAGCTGTCAATGGCATCATCGTAAATATTATCAAGTTGAAGTACAGTCCTGGGCGTTGAAGTGTTAGCACTAACCGAAGCACCGGAAATGGACTTAAACCCGGTCTCGGGAAATCCGGGCCGGGTCTCAGAAACGGCCCCGACCCGAGAAGGAGATAAGCGGAGAGAGTTAGAGGGAGAAACAAAGCGATCGGAAGGAGTAGGAGAGACGAAGTGGTTAGAAGATCGGACGTAGCGGAAGGAATGACCTAGGGTTTCATCCAATGGGTCAGCGGCCGTGAAGATCAAGTCCGGCTGGTCATGTTGAGTCCGGTTAGTCGCCGGTTTGAAGCAAGGGAAGAGGGAGGAGAGTCCACTTCCCATCACATTTGATTCGCCACCTTCCGTTTGGACTTCAAGAAATCAAAAGAAAAAAAAAAATTTCCTTTTCTTTTACCTTTCTTTTGAAATTAAGTTCCGGCAAAGTTTTGAAGAAAA

>yang3.Unigene_125358|len=447| Ricinus communis conserved hypothetical protein

**GGGGGGGGGGGGGGGGAGGGGG**ATGTGTGTTAGTTATTAGCGAAACGTAAGGCTGGAAAAAACTGAAAAAGTGGCGTTTGGGTTTTTGTTCCAAAGAAAAGAGAAAATCATTTTCAGTTGGCAGATATCTCTTTTGGTATTCAAGTTCAGGGAGTGGGACGCAAAGAGAAGATCTCTCATCTCTGGTAAGATAGAGAAAAAAAGAGAAAAGGAAAAGCAAAGAAATTTGAGTGAAGGAAAATAAATGAAAACAAAAATTAAAGAAAGTAGAAAAGTAATTCGTTCCTCGTGTTTAGAGAATCAGGTTCTTCGTGAAATAAAGCACATACAGATAGAGAGATAGAAAAAAAAAAGTGAGAGAGAGAGGAGAAAAATGCGCGCAAAAGAAATCTCAGCACTCAATAGAGAACGCAAAGCGCACACATAAAAGGAGAGAAGAAAAAGAAA

>yang3.Unigene_128202|len=3018|Ricinus communis phospholipid:diacylglycerol acyltransferase 2 (PDAT2)

**GGGGGGTTCGCGGGGGTGGGTGGGTGTTGGGTGGGA**CGGTGGAGAAGCAGCAGAAGTCAAATTCCATACACTTCAGTAACATGGCAAGCAACATTACTGATCTTCTCCAGAAATTTTCCTGCAAAGTACTTGTTTCTGTCATGGTTACAGCAGCAATGGGATTAAGAACGAGACTTCAAAAAATCCTGCGACTTCCTTTGTAGTTAGTTCTGTCTTTGAGCCTCAAAGCCAATCATCTCCCTTTCTTTATGGTAAAATTGTTGATAAGTCTTGATGTAATTACAGACGAAGATTTATTTTCTCGGACATTCTTAATAGATCAGAATGAATCCTGTCACCACCAATTTCAGGACCACTAGCACCTGCAGCAACTCTTAGTACATCTTCAATAAAAGCAACATTTCCCATAATGTCAACATGGGAACCACTCCCTGTACCTCTCCCTTCAAGCAGGCTAGCTGGTGGTTTGTGCTCATACTCCCTCACATATGTAGGACTGCCCGACGGGTTGAACCGGGTTTTCCCTCTCCAACCTTTAGCACACATGTAGCCTGAACTAATCACAGGCACACTTTCATCACCATCAGCAAAGAATACTCCCCTTTTTAAGTTACTGTCCTCTTCTCCATCAACTGAGGCATCGATTCGGAATGGAAGAATCTTGCATCTATCAGTTGCTGATAACTTATAGATGTATGATCTTTCTGTTGGCAGTCCAACCCCATATAAGCAGTATATCTCCAGTTCAGGGGCATTAGGCAACCTTGTCTCTAATGAGTTAGACCAATATTTGTAATGTTCATATTTTGGATCATCAAGATCGTCGGCTATCCCATTTGAGAATTGAGCTTCAAGACGTTGCATCATTTTCGGGGCAACAAATCGAAGCAAATCAAAAGTAGAAGCAACTGTAAAAGCTTTATTTTCCACAAATTTTCGAATATTTTCCCTGTTTATTTCATCATATTCGGTCCAAACCTCTTCACATGATGATCTTGAATTCTCGTTGGTAGCGAATAATTCCTTTGAATCAAAAGAAGGAAGCTGTGAAGATGGCAATTCTGATGCTGCCTTGCTAAAAGAAACTATCCTTCCATACTTAACTGAATCGTTTACTGAAGACACTTCAGGAGACCAATCTAAGTTACCCCAAATAGTCTCTCCTCCTTTAGGCATCATTGAAAATAGTGAATCCCACGTTCGAAGCACCCGCAAGCAATTCTCCATGGTATGAAACCAGGGAGTTTCAGAATCCCAAATGCCAGGAAACCTAGCTCTGAAGTCTGCAGCGTATTTGTTTTCTCCAGAGAGTAGATGAGTTATTGCCTTTGGAGTACCCAGAAATGTCGGACCGATGTTCATGATTGATTTTATGTACTTGTTGCACCACCCTGGACTGCCACCGCCGCCCATTGGCGAAGGTGCTTCAACCCATTTGAGGAAGTACAGAAAATACTGAACCCCCATGGAATGAGGAACCACCACCACTTTCTTATTGCCATTTGCTCCATACATGAACTCTATTTTACTCTTCAATTTACTAAGTGAATGGTCTCGAATCTCTGTATTCTGAAAAGAGATCCTCCAATCATAGGTTGCCATATATAGATTATTCCCTTCGTAGCCAATTTTGGCCAAATTCTCGATTAGAACAGCCCAAACAAAAAAACCCAGGAGCAAAATAGTCAGCTGCAGCCAGCCCTGGGATCGGACGAACTCGGATGCCTGGCGGGTCAAGACCAGTCTCATTGTCCAAAGACATGTGATCCAACCAACATGATGGCCTTCGGAACATTCCACTGAAGGTACCACCCCAAAGCCTCTTTCTGAAAAGACCATGAGCACAAGGTCTGCCTTCCCATAGCTCAAGGCCACCAGTGATAGTGCCTGGCACCAAAATCACTGGATGTTTAGCTGTTAACCCTTCTCTTTTTAATCTGTCTCCTACCGATTCAGGCAATGTAGCCTGCAAACTGTGGTACAAGAACAGAATCAGCCACCAAGTGGTCCATATATACCCAATCATCCGGCAACCGCAGTCTATGCAACTCCATGCTTTTGGCTTCCTTTTTCTCCTTTTATTGTTAATTTCCAACTCGTTTTTGGTATCAGGGTTGACAATTTCTTCTTTCTTGTCATATTTTTGGGAATCAAAAGACTGTTTGATAGTAGAATGGACAATTTCTTCTTTCTTGCCACATTTTTTGGATTCAAAAGATTCAGAATTTTCTGGCTCTACACAGCACAGTTTTCGAAACCGAAGAATTGAAGCCATGATATATTAAGGAAAAAAAAACTCAAATTAAATCACCAACAAGAACAACAGATTTCAGGTAATTAATGTGAGAAACAAGAAAGCAAGAAAAACCCAGAAAGGGAAAATAATAATATATTAGAGTATGGTAGATGAAGAGGTAGCAATGGTGGTGAGTTGTGGTGAAAACAAGGTTATGGGCTATAAGTATCTGTTACAGAAAAAGCAGAAAAACACGTGGGGTGAAGAAGAGAGATGAGATGGGCGTAAACCATTTCTCCTTCTCACGGTGTCCACATCCATAATTCCATTTGCTTTATTAGCATGGAACGATTCAAGAGGATTTGTTTATGCTTACTACATGCTTCGTACAATTTCAGACATTCTAAGTCAAGGAAGCATATTCTGCCTTTCTGTTGCATATGCAGTAAGAATTAGTGTCTACTGTTGAGAAACCATAGACATAAGAATGCAGAGGCATCAATTTTTTCCCCTGCATTTGTTAGGCAGATTTTATAAAGGAAGGCAAGAGGGCTGCAGCTTTTAGTTGGATGACTGGGCTTGTCTCGATTTCTCATCTCTTAGGCAATGTTTTGGCACATTTTCTTCCCGAAAAATTTATTTTTCTCGTATGCAGGTTCTGTGAATTCATGTTCATATTTAAGTCTAGTTTATTTTTATTGGATGACCTGTTTGTTGCTGTGTGTAATGATTGATTATTTGTTTCAGGTTTCAGTTGTTCTCTTGATCTTTTGCCCAGTGTA

>yang3.Unigene_128211|len=2851|Ricinus communis phospholipid:diacylglycerol acyltransferase 2 (PDAT2)

**GGGGGGTTCGCGGGGGTGGGTGGGTGTTGGGTGGGACGG**TGGAGAAGCAGCAGAAGTCAAATTCCATACACTTCAGTAACATGGCAAGCAACATTACTGATCTTCTCCAGAAATTTTCCTGCAAAGTACTTGTTTCTGTCATGGTTACAGCAGCAATGGGATTAAGAACGAGACTTCAAAAAATCCTGCGACTTCCTTTGTAGTTAGTTCTGTCTTTGAGCCTCAAAGCCAATCATCTCCCTTTCTTTATGGTAAAATTGTTGATAAGTCTTGATGTAATTACAGACGAAGATTTATTTTCTCGGACATTCTTAATAGATCAGAATGAATCCTGTCACCACCAATTTCAGGACCACTAGCACCTGCAGCAACTCTTAGTACATCTTCAATAAAAGCAACATTTCCCATAATGTCAACATGGGAACCACTCCCTGTACCTCTCCCTTCAAGCAGGCTAGCTGGTGGTTTGTGCTCATACTCCCTCACATATGTAGGACTGCCCGACGGGTTGAACCGGGTTTTCCCTCTCCAACCTTTAGCACACATGTAGCCTGAACTAATCACAGGCACACTTTCATCACCATCAGCAAAGAATACTCCCCTTTTTAAGTTACTGTCCTCTTCTCCATCAACTGAGGCATCGATTCGGAATGGAAGAATCTTGCATCTATCAGTTGCTGATAACTTATAGATGTATGATCTTTCTGTTGGCAGTCCAACCCCATATAAGCAGTATATCTCCAGTTCAGGGGCATTAGGCAACCTGACAGAAGAAATTCAACTCTCATAAGTTCAAGTGGTGTTTGCCTGATTTCACGACTAACAAAAAATATATGCATTGATGCTCTTACCTTGTCTCTAATGAGTTAGACCAATATTTGTAATGTTCATATTTTGGATCATCAAGATCGTCGGCTATCCCATTTGAGAATTGAGCTTCAAGACGTTGCATCATTTTCGGGGCAACAAATCGAAGCAAATCAAAAGTAGAAGCAACTGTAAAAGCTTTATTTTCCACAAATTTTCGAATATTTTCCCTGTTTATTTCATCATATTCGGTCCAAACCTCTTCACATGATGATCTTGAATTCTCGTTGGTAGCGAATAATTCCTTTGAATCAAAAGAAGGAAGCTGTGAAGATGGCAATTCTGATGCTGCCTTGCTAAAAGAAACTATCCTTCCATACTTAACTGAATCGTTTACTGAAGACACTTCAGGAGACCAATCTAAGTTACCCCAAATAGTCTCTCCTCCTTTAGGCATCATTGAAAATAGTGAATCCCACGTTCGAAGCACCCGCAAGCAATTCTCCATGGTATGAAACCAGGGAGTTTCAGAATCCCAAATGCCAGGAAACCTAGCTCTGAAGTCTGCAGCGTATTTGTTTTCTCCAGAGAGTAGATGAGTTATTGCCTTTGGAGTACCCAGAAATGTCGGACCGATGTTCATGATTGATTTTATGTACTTGTTGCACCACCCTGGACTGCCACCGCCGCCCATTGGCGAAGGTGCTTCAACCCATTTGAGGAAGTACAGAAAATACTGAACCCCCATGGAATGAGGAACCACCACCACTTTCTTATTGCCATTTGCTCCATACATGAACTCTATTTTACTCTTCAATTTACTAAGTGAATGGTCTCGAATCTCTGTATTCTGAAAAGAGATCCTCCAATCATAGGTTGCCATATATAGATTATTCCCTTCGTAGCCAATTTTGGCCAAATTCTCGATTAGAACAGCCCAAACAAAAAAACCCAGGAGCAAAATAGTCAGCTGCAGCCAGCCCTGGGATCGGACGAACTCGGATGCCTGGCGGGTCAAGACCAGTCTCATTGTCCAAAGACATGTGATCCAACCAACATGATGGCCTTCGGAACATTCCACTGAAGGTACCACCCCAAAGCCTCTTTCTGAAAAGACCATGAGCACAAGGTCTGCCTTCCCATAGCTCAAGGCCACCAGTGATAGTGCCTGGCACCAAAATCACTGGATGTTTAGCTGTTAACCCTTCTCTTTTTAATCTGTCTCCTACCGATTCAGGCAATGTAGCCTGCAAACTGTGGTACAAGAACAGAATCAGCCACCAAGTGGTCCATATATACCCAATCATCCGGCAACCGCAGTCTATGCAACTCCATGCTTTTGGCTTCCTTTTTCTCCTTTTATTGTTAATTTCCAACTCGTTTTTGGTATCAGGGTTGACAATTTCTTCTTTCTTGTCATATTTTTGGGAATCAAAAGACTGTTTGATAGTAGAATGGACAATTTCTTCTTTCTTGCCACATTTTTTGGATTCAAAAGATTCAGAATTTTCTGGCTCTACACAGCACAGTTTTCGAAACCGAAGAATTGAAGCCATGATATATTAAGGAAAAAAAAACTCAAATTAAATCACCAACAAGAACAACAGATTTCAGGTAATTAATGTGAGAAACAAGAAAGCAAGAAAAACCCAGAAAGGGAAAATAATAATATATTAGAGTATGGTAGATGAAGAGGTAGCAATGGTGGTGAGTTGTGGTGAAAACAAGGTTATGGGCTATAAGTATCTGTTACAGAAAAAGCAGAAAAACACGTGGGGTGAAGAAGAGAGATGAGATGGGCGTAAACCATTTCTCCTTCTCACGGTGTCCACATCCATAATTCCATTTGCTTTATTAGCATGGAACGATTCAAGAGGATTTGTTTATGCTTACTACATGCTTCGTACAATTTCAGACATTCTAAGTCAAGGAAGCATATTCTGCCTTTCTGTTGCATATGCAGTAAGAATTAGTGTCTACTGTTGAGAAACCATAGACATAAGAATGCAGAGGCATCAATTTTTTCCCCTGCATTTGTTAGGCA

>yang3.Unigene_128217|len=2778|Ricinus communis phospholipid:diacylglycerol acyltransferase 2 (PDAT2)

**GGGGGGTTCGCGGGGGTGGGTGGG**TGTTGGGTGGGACGGTGGAGAAGCAGCAGAAGTCAAATTCCATACACTTCAGTAACATGGCAAGCAACATTACTGATCTTCTCCAGAAATTTTCCTGCAAAGTACTTGTTTCTGTCATGGTTACAGCAGCAATGGGATTAAGAACGAGACTTCAAAAAATCCTGCGACTTCCTTTGTAGTTAGTTCTGTCTTTGAGCCTCAAAGCCAATCATCTCCCTTTCTTTATGGTAAAATTGTTGATAAGTCTTGATGTAATTACAGACGAAGATTTATTTTCTCGGACATTCTTAATAGATCAGAATGAATCCTGTCACCACCAATTTCAGGACCACTAGCACCTGCAGCAACTCTTAGTACATCTTCAATAAAAGCAACATTTCCCATAATGTCAACATGGGAACCACTCCCTGTACCTCTCCCTTCAAGCAGGCTAGCTGGTGGTTTGTGCTCATACTCCCTCACATATGTAGGACTGCCCGACGGGTTGAACCGGGTTTTCCCTCTCCAACCTTTAGCACACATGTAGCCTGAACTAATCACAGGCACACTTTCATCACCATCAGCAAAGAATACTCCCCTTTTTAAGTTACTGTCCTCTTCTCCATCAACTGAGGCATCGATTCGGAATGGAAGAATCTTGCATCTATCAGTTGCTGATAACTTATAGATGTATGATCTTTCTGTTGGCAGTCCAACCCCATATAAGCAGTATATCTCCAGTTCAGGGGCATTAGGCAACCTGACAGAAGAAATTCAACTCTCATAAGTTCAAGTGGTGTTTGCCTGATTTCACGACTAACAAAAAATATATGCATTGATGCTCTTACCTTGTCTCTAATGAGTTAGACCAATATTTGTAATGTTCATATTTTGGATCATCAAGATCGTCGGCTATCCCATTTGAGAATTGAGCTTCAAGACGTTGCATCATTTTCGGGGCAACAAATCGAAGCAAATCAAAAGTAGAAGCAACTGTAAAAGCTTTATTTTCCACAAATTTTCGAATATTTTCCCTGTTTATTTCATCATATTCGGTCCAAACCTCTTCACATGATGATCTTGAATTCTCGTTGGTAGCGAATAATTCCTTTGAATCAAAAGAAGGAAGCTGTGAAGATGGCAATTCTGATGCTGCCTTGCTAAAAGAAACTATCCTTCCATACTTAACTGAATCGTTTACTGAAGACACTTCAGGAGACCAATCTAAGTTACCCCAAATAGTCTCTCCTCCTTTAGGCATCATTGAAAATAGTGAATCCCACGTTCGAAGCACCCGCAAGCAATTCTCCATGGTATGAAACCAGGGAGTTTCAGAATCCCAAATGCCAGGAAACCTAGCTCTGAAGTCTGCAGCGTATTTGTTTTCTCCAGAGAGTAGATGAGTTATTGCCTTTGGAGTACCCAGAAATGTCGGACCGATGTTCATGATTGATTTTATGTACTTGTTGCACCACCCTGGACTGCCACCGCCGCCCATTGGCGAAGGTGCTTCAACCCATTTGAGGAAGTACAGAAAATACTGAACCCCCATGGAATGAGGAACCACCACCACTTTCTTATTGCCATTTGCTCCATACATGAACTCTATTTTACTCTTCAATTTACTAAGTGAATGGTCTCGAATCTCTGTATTCTGAAAAGAGATCCTCCAATCATAGGTTGCCATATATAGATTATTCCCTTCGTAGCCAATTTTGGCCAAATTCTCGATTAGAACAGCCCAAACAAAAAAACCCAGGAGCAAAATAGTCAGCTGCAGCCAGCCCTGGGATCGGACGAACTCGGATGCCTGGCGGGTCAAGACCAGTCTCATTGTCCAAAGACATGTGATCCAACCAACACGATGGCCTTTTGAACAGTTCACTGAAGGTACCACCCCAAAGTTTCTGCCTGAAAAGACCATGTGCACAAGGTTTGCCTTCCCATAGCTCAAGCCCACCAGTGATAATGCCAGGCACCAAAACCACTGGATGTTTAGCTGTTAAGCCTTCTCTTTTTAATCTTTCTCCTGGCGATTCAGGGACCTTAAATCCAGGCAATGCGGCAGGCAAACTGTAATACAAGAACAGCAGCAGCCACCAAGCGGTGCACACACACCCAATCATCCGACAGAAGCAGTCTATGGAACACCATGCCTTTGGGTTTCTTTTTCTCCTTTTCTTGTTATTTTCTGACTCATTTTTAGCATCAGGGGTAACAATTTCTTCTTTCTTGTCAAATTTTTTGGATTCAAAAGATTCTGAATTTACCTGTTCTACATAGCATAACTTTCGAAATCGAAGAATTAAAGCCATGATTGATTTATAAAGCTTCAAAATCACCGCCAAAAACAACCCATTTCAGGAATTTGAGAAAAAGAACAGATTGAGGTGCGAAACGAGAAAGCAAGAAAAACCCAGAAAGGGAAAATAATAATATATTAGAGTATGGTAGATGAAGAGGTAGCAATGGTGGTGAGTTGTGGTGAAAACAAGGTTATGGGCTATAAGTATCTGTTACAGAAAAAGCAGAAAAACACGTGGGGTGAAGAAGAGAGATGAGATGGGCGTAAACCATTTCTCCTTCTCACGGTGTCCACATCCATAATTCCATTTGGTATGTTTCTGTTGGATAGTCTCAAATCAAGAACTAGAATTTATTCTTAATGTTCTCAAATTATGTTTCTGAAATCACACGATTCGAACATAAAAGTGAAGAATGCAATAGTATTTGTCTGGATGTCGAAGACACTTAAGTAGGTTAGAGTTAGACCAA

>yang3.Unigene_128227|len=2690|Ricinus communis phospholipid:diacylglycerol acyltransferase 2 (PDAT2)

**GGGGGGTTCGCGGGGGTGGGTGGGTGTTGGGTGGGACGGTGG**AGAAGCAGCAGAAGTCAAATTCCATACACTTCAGTAACATGGCAAGCAACATTACTGATCTTCTCCAGAAATTTTCCTGCAAAGTACTTGTTTCTGTCATGGTTACAGCAGCAATGGGATTAAGAACGAGACTTCAAAAAATCCTGCGACTTCCTTTGTAGTTAGTTCTGTCTTTGAGCCTCAAAGCCAATCATCTCCCTTTCTTTATGGTAAAATTGTTGATAAGTCTTGATGTAATTACAGACGAAGATTTATTTTCTCGGACATTCTTAATAGATCAGAATGAATCCTGTCACCACCAATTTCAGGACCACTAGCACCTGCAGCAACTCTTAGTACATCTTCAATAAAAGCAACATTTCCCATAATGTCAACATGGGAACCACTCCCTGTACCTCTCCCTTCAAGCAGGCTAGCTGGTGGTTTGTGCTCATACTCCCTCACATATGTAGGACTGCCCGACGGGTTGAACCGGGTTTTCCCTCTCCAACCTTTAGCACACATGTAGCCTGAACTAATCACAGGCACACTTTCATCACCATCAGCAAAGAATACTCCCCTTTTTAAGTTACTGTCCTCTTCTCCATCAACTGAGGCATCGATTCGGAATGGAAGAATCTTGCATCTATCAGTTGCTGATAACTTATAGATGTATGATCTTTCTGTTGGCAGTCCAACCCCATATAAGCAGTATATCTCCAGTTCAGGGGCATTAGGCAACCTTGTCTCTAATGAGTTAGACCAATATTTGTAATGTTCATATTTTGGATCATCAAGATCGTCGGCTATCCCATTTGAGAATTGAGCTTCAAGACGTTGCATCATTTTCGGGGCAACAAATCGAAGCAAATCAAAAGTAGAAGCAACTGTAAAAGCTTTATTTTCCACAAATTTTCGAATATTTTCCCTGTTTATTTCATCATATTCGGTCCAAACCTCTTCACATGATGATCTTGAATTCTCGTTGGTAGCGAATAATTCCTTTGAATCAAAAGAAGGAAGCTGTGAAGATGGCAATTCTGATGCTGCCTTGCTAAAAGAAACTATCCTTCCATACTTAACTGAATCGTTTACTGAAGACACTTCAGGAGACCAATCTAAGTTACCCCAAATAGTCTCTCCTCCTTTAGGCATCATTGAAAATAGTGAATCCCACGTTCGAAGCACCCGCAAGCAATTCTCCATGGTATGAAACCAGGGAGTTTCAGAATCCCAAATGCCAGGAAACCTAGCTCTGAAGTCTGCAGCGTATTTGTTTTCTCCAGAGAGTAGATGAGTTATTGCCTTTGGAGTACCCAGAAATGTCGGACCGATGTTCATGATTGATTTTATGTACTTGTTGCACCACCCTGGACTGCCACCGCCGCCCATTGGCGAAGGTGCTTCAACCCATTTGAGGAAGTACAGAAAATACTGAACCCCCATGGAATGAGGAACCACCACCACTTTCTTATTGCCATTTGCTCCATACATGAACTCTATTTTACTCTTCAATTTACTAAGTGAATGGTCTCGAATCTCTGTATTCTGAAAAGAGATCCTCCAATCATAGGTTGCCATATATAGATTATTCCCTTCGTAGCCAATTTTGGCCAAATTCTCGATTAGAACAGCCCAAACAAAAAAACCCAGGAGCAAAATAGTCAGCTGCAGCCAGCCCTGGGATCGGACGAACTCGGATGCCTGGCGGGTCAAGACCAGTCTCATTGTCCAAAGACATGTGATCCAACCAACACGATGGCCTTTTGAACAGTTCACTGAAGGTACCACCCCAAAGTTTCTGCCTGAAAAGACCATGTGCACAAGGTTTGCCTTCCCATAGCTCAAGCCCACCAGTGATAATGCCAGGCACCAAAACCACTGGATGTTTAGCTGTTAAGCCTTCTCTTTTTAATCTTTCTCCTGGCGATTCAGGGACCTTAAATCCAGGCAATGCGGCAGGCAAACTGTAATACAAGAACAGCAGCAGCCACCAAGCGGTGCACACACACCCAATCATCCGACAGAAGCAGTCTATGGAACACCATGCCTTTGGGTTTCTTTTTCTCCTTTTCTTGTTATTTTCTGACTCATTTTTAGCATCAGGGGTAACAATTTCTTCTTTCTTGTCAAATTTTTTGGATTCAAAAGATTCTGAATTTACCTGTTCTACATAGCATAACTTTCGAAATCGAAGAATTAAAGCCATGATTGATTTATAAAGCTTCAAAATCACCGCCAAAAACAACCCATTTCAGGAATTTGAGAAAAAGAACAGATTGAGGTGCGAAACGAGAAAGCAAGAAAAACCCAGAAAGGGAAAATAATAATATATTAGAGTATGGTAGATGAAGAGGTAGCAATGGTGGTGAGTTGTGGTGAAAACAAGGTTATGGGCTATAAGTATCTGTTACAGAAAAAGCAGAAAAACACGTGGGGTGAAGAAGAGAGATGAGATGGGCGTAAACCATTTCTCCTTCTCACGGTGTCCACATCCATAATTCCATTTGGTATGTTTCTGTTGGATAGTCTCAAATCAAGAACTAGAATTTATTCTTAATGTTCTCAAATTATGTTTCTGAAATCACACGATTCGAACATAAAAGTGAAGAATGCAATAGTATTTGTCTGGATGTCGAAGACACTTAAGTAGGTTAGAGTTAGACCAA

>yang3.Unigene_147664|Populus trichocarpa bifunctional nuclease family protein

**GGGGGGGGGGGGGAAGGGGGGGGCGGGGAGGATAAGGTG**GAGTTGGTGATGGTGAGGGACTACTCCGGTGGAGGAGAGACCGGAAATGAGGGAAGGAAGGAAGGGAGATGATCAGAGTGTAGACTAAAACAGATTGCTTAGGAGAAAGATGGATATATACCTGAGCAATTTTGTAGACTATGATGTGGCCATTAGTGCCCCGGCCATGAATCACTGGAAACACAA

>yang3.Unigene_131907|len=255| PREDICTED:Glycine max histone-lysine N-methyltransferase 2D-like

**CCCTGGCCCCCCCCCCCCACACCCTTACCCCCCCC**AGCTAACCCAGCCAGTGAATCCTCATCTCCAGCCTCAGCCACAGCATTCATCAGCCCATGCAGTGACTGGTCATCATTCTTATCCACAACCGCAGTCTCTACAGCAAATGCAGCCGGGAGGCCAACAGCATCCTGCACATTTGTATCCTCAAGGTGGGCTTCAACCACAGTCACAATATTCTGTGCAAATGCAGAATCAATTACTTCAACGACCTCCTCT

>yang3.Unigene_110133|len=680| Ricinus communis 3-5 exonuclease

CCCAACTACCCACCCACACCCACAAAACAAAATATAAATAAATAATAAAAAAACACCACAACCACCATCTCACTCCCAGATCTAAATTTTGTCTGCCATGGGTGGTGTCCCATTAGCTGATGGAACCAGAATGCTACCACCTCATTGTGCGAAATAAATTAAGCATACGAGTAACTAAAATCTTAGAGTTTCATAATGCCTAAAAAAGAACAAAGTAAACGGAGACTTTCAGTTCCAGGTCTCCACAATCGATTTCAAGGGCTTAAACTTGCAAGATGATGAAAGATGGTTCATCCCCCAGTTTACATGTGCTTACCAAGTTTAAGAATTCTAGGAGACTGCAAAATACGAGTTAGGCAGTTGTCCAAAATACTGGATACATCTTCAAATAACTTTATCAAGTCAAATATAAAAACCATCTTATCAGAAGCAATTTGCATGATAGAAACCTTGTTTGGTTTGCTACCTTTCTCATAATTAGGCTTCCATTCACAATCCATGCCCACAACTTTACATCCCTCAATGTGGCATGTTGCATCACATAGCACACCAACTTCATCAACCCAAACAATATCTTCAACACCTAACTCATTAAGTTGCAGATTTCTGTCGTGTGCAAGAGTTGCCTCAAGTTCTTTGCCTTTCAGAAAACCTTCAAGTGAGTAGCGATCACAAAGTTC

>yang3.Unigene_45401|len=1464| Ricinus communis DNA binding protein

**CCCCCCCCTCCGCCCCCCCCCCCTCC**TTTTTATTTTATCTTCTCCTTCGAACCACCATTTTCCATTGATCTTGTACCTTAATCAATGAACCAAGAAGAAGAAAAATCCCCAAAATCTCCTCCTAAAGCCACCTCATCTACTTCTGGACCAGAGACGACGCGGCCCTTATTGCAGCCATTAATGGGTTCTCAATTGTTTCCTTCTGGGTCTTTTAATGTGGAGGATTTGAACATTTCTGGGTCTCGTTTTTCTGCTTCGTTCCATTCTCCATTAATGGAATTTGAAGCGTTCTCCGCCATCGAACAATCTGTTTGTTCTCCTTCGCAGATGTTTGAATTCAAAGCGAATCCATTATCTGAAATAGCTTCGCCTGGAGGAGGAGATACAGGTGATGATGGGGAAGAAAACATTGGGGTGCCTCAGCCTCTTGCTAGTTTGCAAGAGAATCCAATTCCGCCGTTTCTCTCAAAAACTTATGATCTGGTGAATGATCGGACGATGGACCCTATAATCTCATGGGGCTCCGCCGGAGAGAGCTTCGTGGTGTGGGACCCGGTGGAATTCGCTAGAGTCGTGCTACCTAGGAATTTCAAGCACAACAATTTCTCCAGTTTTGTTCGGCAACTCAATACTTATGTGGGTATTAATTGCGGTATCACAATAGCCTTCATTGGCTGCTGTGTTACTTCCTTTCTGGATATTTGTTCGTTTGTTGATCTTTTCCATTTTTGGGTTATTTTTACTGTTTCTTATATTGTATTTGGATCTGACATTTTCAATCCTTGTATATGTAGCTGATGAAGTTTTTTGTACTAACATTGCGGCCATGAGTATATAAGTGAGGTTGCTGGAATTTGGTTGGTGCATTCTTTAAATATTTGATAATTTGCACGGGATTATGTAATGGAGTGTATCTGATTTTATGTATTTGTACTAAGTTAATTTTTGTAATTTTTCTGTCAATGTAATTGTAAAGCTTTCAGTTGTTTCTTAGTGAATTATAATGGGTTTCTGGGTTACCTTAGAGTTGTAATGTTGACTTGTTGGGGCATAATAGTCTCTGTAGGGCTTGTCTCCTCTATGAAATGATTACTGTAGATTTTAGAAAACCATAAGGGTTCTGCCTTCAGGTGATAGATAGTACCTGAGGTTTTGATCTTCATACTGCCAACAAAAGGACATCCTTCCCCTCTGAGCAAAGTATTGGAGCGGGGAGATACATCTTGTGCAGGGATTCCGCAAGATTGATAGCGATAAATGGGAATTTGCCAATGAAGCTTTTCGACGAGATCAGAGGCATCTGTTAAAAAACATTCAGAGACGCAGAACACTGCAATCCCAGCAGATTGGGAACTATACAAGACCTACTGAAGCAGGGAAGTCAGAACTGGAAAGTGAGATAGAAACATTGAGGAAAGAGAGGAGTGTGATGATGCGGGAGGTTGTGGAACTGCAGCAGCAGCA

>yang3.Unigene_45402|len=1431| Ricinus communis DNA binding protein

**CCCCCCCCTCCGCCCCCCCCCCCTCC**TTTTTATTTTATCTTCTCCTTCGAACCACCATTTTCCATTGATCTTGTACCTTAATCAATGAACCAAGAAGAAGAAAAATCCCCAAAATCTCCTCATAAAGTCACCTTATCTGCTTCGGGACCAGAGACGACGAGGCCCTTATTGCAGCCATTAATTGGTTCTCAGGTGTTTCCTTCTGGGGTTTTTAACGTGGAGGTTGCGAACATTTCTAGGTCTCCTTTCTCTGCTTCGTTCCCTTCTCCTTTAATGGAATTTGAAGCATTCTCCGCCATCGAACAATCTGTTTGTTCTCCTTCGCAGATGTTTGAATCGCCTGGAGGAGATACAGGTGATGATGGGGAAGAAAACATTGGGGTGCCTCAGCCTCTTGCTAGTTTGCAAGAGAATCCAATTCCGCCGTTTCTCTCAAAAACTTATGATCTGGTGAATGATCGGACGATGGACCCTATAATCTCATGGGGCTCCGCCGGAGAGAGCTTCGTGGTGTGGGACCCGGTGGAATTCGCTAGAGTCGTGCTACCTAGGAATTTCAAGCACAACAATTTCTCCAGTTTTGTTCGGCAACTCAATACTTATGTGGGTATTAATTGCGGTATCACAATAGCCTTCATTGGCTGCTGTGTTACTTCCTTTCTGGATATTTGTTCGTTTGTTGATCTTTTCCATTTTTGGGTTATTTTTACTGTTTCTTATATTGTATTTGGATCTGACATTTTCAATCCTTGTATATGTAGCTGATGAAGTTTTTTGTACTAACATTGCGGCCATGAGTATATAAGTGAGGTTGCTGGAATTTGGTTGGTGCATTCTTTAAATATTTGATAATTTGCACGGGATTATGTAATGGAGTGTATCTGATTTTATGTATTTGTACTAAGTTAATTTTTGTAATTTTTCTGTCAATGTAATTGTAAAGCTTTCAGTTGTTTCTTAGTGAATTATAATGGGTTTCTGGGTTACCTTAGAGTTGTAATGTTGACTTGTTGGGGCATAATAGTCTCTGTAGGGCTTGTCTCCTCTATGAAATGATTACTGTAGATTTTAGAAAACCATAAGGGTTCTGCCTTCAGGTGATAGATAGTACCTGAGGTTTTGATCTTCATACTGCCAACAAAAGGACATCCTTCCCCTCTGAGCAAAGTATTGGAGCGGGGAGATACATCTTGTGCAGGGATTCCGCAAGATTGATAGCGATAAATGGGAATTTGCCAATGAAGCTTTTCGACGAGATCAGAGGCATCTGTTAAAAAACATTCAGAGACGCAGAACACTGCAATCCCAGCAGATTGGGAACTATACAAGACCTACTGAAGCAGGGAAGTCAGAACTGGAAAGTGAGATAGAAACATTGAGGAAAGAGAGGAGTGTGATGATGCGGGAGGTTGTGGAACTGCAGCAGCAGCA

>yang3.Unigene_45403|len=1382| Ricinus communis DNA binding protein

**CCCCCCCCTCCGCCCCCCCCCCCTCC**TTTTTATTTTATCTTCTCCTTCGAACCACCATTTTCCATTGATCTTGTACCTTAATCAATGAACCAAGAAGAAGAAAAATCCCCAAAATCTCCTCCTAAAGCCACCTCATCTACTTCTGGACCAGAGACGACGCGGCCCTTATTGCAGCCATTAATGGGTTCTCAATTGTTTCCTTCTGGGTCTTTTAATGTGGAGGATTTGAACATTTCTGGGTCTCGTTTTTCTGCTTCGTTCCATTCTCCATTAATGGAATTTGAAGCGTTCTCCGCCATCGAACAATCTGTTTGTTCTCCTTCGCAGATGTTTGAATTCAAAGCGAATCCATTATCTGAAATAGCTTCGCCTGGAGGAGGAGATACAGGTGATGATGGGGAAGAAAACATTGGGGTGCCTCAGCCTCTTGCTAGTTTGCAAGAGAATCCAATTCCGCCGTTTCTCTCAAAAACTTATGATCTGGTGAATGATCGGACGATGGACCCTATAATCTCATGGGGCTCCGCCGGAGAGAGCTTCGTGGTGTGGGACCCGGTGGAATTCGCTAGAGTCGTGCTACCTAGGAATTTCAAGCACAACAATTTCTCCAGTTTTGTTCGGCAACTCAATACTTATGTGGGTATTAATTGCGGTATCACAATAGCCTTCATTGGCTGCTGTGTTACTTCCTTTCTGGATATTTGTTCGTTTGTTGATCTTTTCCATTTTTGGGTTATTTTTACTGTTTCTTATATTGTATTTGGATCTGACATTTTCAATCCTTGTATATGTAGCTGATGAAGTTTTTTGTACTAACATTGCGGCCATGAGTATATAAGTGAGGTTGCTGGAATTTGGTTGGTGCATTCTTTAAATATTTGATAATTTGCACGGGATTATGTAATGGAGTGTATCTGATTTTATGTATTTGTACTAAGTTAATTTTTGTAATTTTTCTGTCAATGTAATTGTAAAGCTTTCAGTTGTTTCTTAGTGAATTATAATGGGTTTCTGGGTTACCTTAGAGTTGTAATGTTGACTTGTTGGGGCATAATAGTCTCTGTAGGGCTTGTCTCCTCTATGAAATGATTACTGTAGATTTTAGAAAACCATAAGGGTTCTGCCTTCAGGTGATAGATAGTACCTGAGGTTTTGATCTTCATACTGCCAACAAAAGGACATCCTTCCCCTCTGAGCAAAGTATTGGAGCGGGGAGATACATCTTGTGCAGGTAGAATTTAAGGTTCTGGTGGAGCATTTTATTCCTATTTGACAGTGTGAAGACTCTTTATTTTCTGCATGTAATGTAAATCCATGGCTGTGACATTTTTCTTATGCAAATCAACTCTTTTGCGTAAAAATTGACAGCTTTTATGTTTTCT

>yang3.Unigene_45404|len=1349| Ricinus communis DNA binding protein

**CCCCCCCCTCCGCCCCCCCCCCCTCC**TTTTTATTTTATCTTCTCCTTCGAACCACCATTTTCCATTGATCTTGTACCTTAATCAATGAACCAAGAAGAAGAAAAATCCCCAAAATCTCCTCATAAAGTCACCTTATCTGCTTCGGGACCAGAGACGACGAGGCCCTTATTGCAGCCATTAATTGGTTCTCAGGTGTTTCCTTCTGGGGTTTTTAACGTGGAGGTTGCGAACATTTCTAGGTCTCCTTTCTCTGCTTCGTTCCCTTCTCCTTTAATGGAATTTGAAGCATTCTCCGCCATCGAACAATCTGTTTGTTCTCCTTCGCAGATGTTTGAATCGCCTGGAGGAGATACAGGTGATGATGGGGAAGAAAACATTGGGGTGCCTCAGCCTCTTGCTAGTTTGCAAGAGAATCCAATTCCGCCGTTTCTCTCAAAAACTTATGATCTGGTGAATGATCGGACGATGGACCCTATAATCTCATGGGGCTCCGCCGGAGAGAGCTTCGTGGTGTGGGACCCGGTGGAATTCGCTAGAGTCGTGCTACCTAGGAATTTCAAGCACAACAATTTCTCCAGTTTTGTTCGGCAACTCAATACTTATGTGGGTATTAATTGCGGTATCACAATAGCCTTCATTGGCTGCTGTGTTACTTCCTTTCTGGATATTTGTTCGTTTGTTGATCTTTTCCATTTTTGGGTTATTTTTACTGTTTCTTATATTGTATTTGGATCTGACATTTTCAATCCTTGTATATGTAGCTGATGAAGTTTTTTGTACTAACATTGCGGCCATGAGTATATAAGTGAGGTTGCTGGAATTTGGTTGGTGCATTCTTTAAATATTTGATAATTTGCACGGGATTATGTAATGGAGTGTATCTGATTTTATGTATTTGTACTAAGTTAATTTTTGTAATTTTTCTGTCAATGTAATTGTAAAGCTTTCAGTTGTTTCTTAGTGAATTATAATGGGTTTCTGGGTTACCTTAGAGTTGTAATGTTGACTTGTTGGGGCATAATAGTCTCTGTAGGGCTTGTCTCCTCTATGAAATGATTACTGTAGATTTTAGAAAACCATAAGGGTTCTGCCTTCAGGTGATAGATAGTACCTGAGGTTTTGATCTTCATACTGCCAACAAAAGGACATCCTTCCCCTCTGAGCAAAGTATTGGAGCGGGGAGATACATCTTGTGCAGGTAGAATTTAAGGTTCTGGTGGAGCATTTTATTCCTATTTGACAGTGTGAAGACTCTTTATTTTCTGCATGTAATGTAAATCCATGGCTGTGACATTTTTCTTATGCAAATCAACTCTTTTGCGTAAAAATTGACAGCTTTTATGTTTTCT

>yang3.Unigene_45405|len=1306| Ricinus communis DNA binding protein

**CCCCCCCCTCCGCCCCCCCCCCCTCC**TTTTTATTTTATCTTCTCCTTCGAACCACCATTTTCCATTGATCTTGTACCTTAATCAATGAACCAAGAAGAAGAAAAATCCCCAAAATCTCCTCCTAAAGCCACCTCATCTACTTCTGGACCAGAGACGACGCGGCCCTTATTGCAGCCATTAATGGGTTCTCAATTGTTTCCTTCTGGGTCTTTTAATGTGGAGGATTTGAACATTTCTGGGTCTCGTTTTTCTGCTTCGTTCCATTCTCCATTAATGGAATTTGAAGCGTTCTCCGCCATCGAACAATCTGTTTGTTCTCCTTCGCAGATGTTTGAATTCAAAGCGAATCCATTATCTGAAATAGCTTCGCCTGGAGGAGGAGATACAGGTGATGATGGGGAAGAAAACATTGGGGTGCCTCAGCCTCTTGCTAGTTTGCAAGAGAATCCAATTCCGCCGTTTCTCTCAAAAACTTATGATCTGGTGAATGATCGGACGATGGACCCTATAATCTCATGGGGCTCCGCCGGAGAGAGCTTCGTGGTGTGGGACCCGGTGGAATTCGCTAGAGTCGTGCTACCTAGGAATTTCAAGCACAACAATTTCTCCAGTTTTGTTCGGCAACTCAATACTTATCTGATGAAGTTTTTTGTACTAACATTGCGGCCATGAGTATATAAGTGAGGTTGCTGGAATTTGGTTGGTGCATTCTTTAAATATTTGATAATTTGCACGGGATTATGTAATGGAGTGTATCTGATTTTATGTATTTGTACTAAGTTAATTTTTGTAATTTTTCTGTCAATGTAATTGTAAAGCTTTCAGTTGTTTCTTAGTGAATTATAATGGGTTTCTGGGTTACCTTAGAGTTGTAATGTTGACTTGTTGGGGCATAATAGTCTCTGTAGGGCTTGTCTCCTCTATGAAATGATTACTGTAGATTTTAGAAAACCATAAGGGTTCTGCCTTCAGGTGATAGATAGTACCTGAGGTTTTGATCTTCATACTGCCAACAAAAGGACATCCTTCCCCTCTGAGCAAAGTATTGGAGCGGGGAGATACATCTTGTGCAGGGATTCCGCAAGATTGATAGCGATAAATGGGAATTTGCCAATGAAGCTTTTCGACGAGATCAGAGGCATCTGTTAAAAAACATTCAGAGACGCAGAACACTGCAATCCCAGCAGATTGGGAACTATACAAGACCTACTGAAGCAGGGAAGTCAGAACTGGAAAGTGAGATAGAAACATTGAGGAAAGAGAGGAGTGTGATGATGCGGGAGGTTGTGGAACTGCAGCAGCAGCA

>yang3.Unigene_45406|len=1273| Ricinus communis DNA binding protein

**CCCCCCCCTCCGCCCCCCCCCCCTCC**TTTTTATTTTATCTTCTCCTTCGAACCACCATTTTCCATTGATCTTGTACCTTAATCAATGAACCAAGAAGAAGAAAAATCCCCAAAATCTCCTCATAAAGTCACCTTATCTGCTTCGGGACCAGAGACGACGAGGCCCTTATTGCAGCCATTAATTGGTTCTCAGGTGTTTCCTTCTGGGGTTTTTAACGTGGAGGTTGCGAACATTTCTAGGTCTCCTTTCTCTGCTTCGTTCCCTTCTCCTTTAATGGAATTTGAAGCATTCTCCGCCATCGAACAATCTGTTTGTTCTCCTTCGCAGATGTTTGAATCGCCTGGAGGAGATACAGGTGATGATGGGGAAGAAAACATTGGGGTGCCTCAGCCTCTTGCTAGTTTGCAAGAGAATCCAATTCCGCCGTTTCTCTCAAAAACTTATGATCTGGTGAATGATCGGACGATGGACCCTATAATCTCATGGGGCTCCGCCGGAGAGAGCTTCGTGGTGTGGGACCCGGTGGAATTCGCTAGAGTCGTGCTACCTAGGAATTTCAAGCACAACAATTTCTCCAGTTTTGTTCGGCAACTCAATACTTATCTGATGAAGTTTTTTGTACTAACATTGCGGCCATGAGTATATAAGTGAGGTTGCTGGAATTTGGTTGGTGCATTCTTTAAATATTTGATAATTTGCACGGGATTATGTAATGGAGTGTATCTGATTTTATGTATTTGTACTAAGTTAATTTTTGTAATTTTTCTGTCAATGTAATTGTAAAGCTTTCAGTTGTTTCTTAGTGAATTATAATGGGTTTCTGGGTTACCTTAGAGTTGTAATGTTGACTTGTTGGGGCATAATAGTCTCTGTAGGGCTTGTCTCCTCTATGAAATGATTACTGTAGATTTTAGAAAACCATAAGGGTTCTGCCTTCAGGTGATAGATAGTACCTGAGGTTTTGATCTTCATACTGCCAACAAAAGGACATCCTTCCCCTCTGAGCAAAGTATTGGAGCGGGGAGATACATCTTGTGCAGGGATTCCGCAAGATTGATAGCGATAAATGGGAATTTGCCAATGAAGCTTTTCGACGAGATCAGAGGCATCTGTTAAAAAACATTCAGAGACGCAGAACACTGCAATCCCAGCAGATTGGGAACTATACAAGACCTACTGAAGCAGGGAAGTCAGAACTGGAAAGTGAGATAGAAACATTGAGGAAAGAGAGGAGTGTGATGATGCGGGAGGTTGTGGAACTGCAGCAGCAGCA

>yang3.Unigene_45407|len=1224| Ricinus communis DNA binding protein

**CCCCCCCCTCCGCCCCCCCCCCCTCC**TTTTTATTTTATCTTCTCCTTCGAACCACCATTTTCCATTGATCTTGTACCTTAATCAATGAACCAAGAAGAAGAAAAATCCCCAAAATCTCCTCCTAAAGCCACCTCATCTACTTCTGGACCAGAGACGACGCGGCCCTTATTGCAGCCATTAATGGGTTCTCAATTGTTTCCTTCTGGGTCTTTTAATGTGGAGGATTTGAACATTTCTGGGTCTCGTTTTTCTGCTTCGTTCCATTCTCCATTAATGGAATTTGAAGCGTTCTCCGCCATCGAACAATCTGTTTGTTCTCCTTCGCAGATGTTTGAATTCAAAGCGAATCCATTATCTGAAATAGCTTCGCCTGGAGGAGGAGATACAGGTGATGATGGGGAAGAAAACATTGGGGTGCCTCAGCCTCTTGCTAGTTTGCAAGAGAATCCAATTCCGCCGTTTCTCTCAAAAACTTATGATCTGGTGAATGATCGGACGATGGACCCTATAATCTCATGGGGCTCCGCCGGAGAGAGCTTCGTGGTGTGGGACCCGGTGGAATTCGCTAGAGTCGTGCTACCTAGGAATTTCAAGCACAACAATTTCTCCAGTTTTGTTCGGCAACTCAATACTTATCTGATGAAGTTTTTTGTACTAACATTGCGGCCATGAGTATATAAGTGAGGTTGCTGGAATTTGGTTGGTGCATTCTTTAAATATTTGATAATTTGCACGGGATTATGTAATGGAGTGTATCTGATTTTATGTATTTGTACTAAGTTAATTTTTGTAATTTTTCTGTCAATGTAATTGTAAAGCTTTCAGTTGTTTCTTAGTGAATTATAATGGGTTTCTGGGTTACCTTAGAGTTGTAATGTTGACTTGTTGGGGCATAATAGTCTCTGTAGGGCTTGTCTCCTCTATGAAATGATTACTGTAGATTTTAGAAAACCATAAGGGTTCTGCCTTCAGGTGATAGATAGTACCTGAGGTTTTGATCTTCATACTGCCAACAAAAGGACATCCTTCCCCTCTGAGCAAAGTATTGGAGCGGGGAGATACATCTTGTGCAGGTAGAATTTAAGGTTCTGGTGGAGCATTTTATTCCTATTTGACAGTGTGAAGACTCTTTATTTTCTGCATGTAATGTAAATCCATGGCTGTGACATTTTTCTTATGCAAATCAACTCTTTTGCGTAAAAATTGACAGCTTTTATGTTTTCT

>yang3.Unigene_45408|len=1191|Ricinus communis DNA binding protein

**CCCCCCCCTCCGCCCCCCCCCCCTCC**TTTTTATTTTATCTTCTCCTTCGAACCACCATTTTCCATTGATCTTGTACCTTAATCAATGAACCAAGAAGAAGAAAAATCCCCAAAATCTCCTCATAAAGTCACCTTATCTGCTTCGGGACCAGAGACGACGAGGCCCTTATTGCAGCCATTAATTGGTTCTCAGGTGTTTCCTTCTGGGGTTTTTAACGTGGAGGTTGCGAACATTTCTAGGTCTCCTTTCTCTGCTTCGTTCCCTTCTCCTTTAATGGAATTTGAAGCATTCTCCGCCATCGAACAATCTGTTTGTTCTCCTTCGCAGATGTTTGAATCGCCTGGAGGAGATACAGGTGATGATGGGGAAGAAAACATTGGGGTGCCTCAGCCTCTTGCTAGTTTGCAAGAGAATCCAATTCCGCCGTTTCTCTCAAAAACTTATGATCTGGTGAATGATCGGACGATGGACCCTATAATCTCATGGGGCTCCGCCGGAGAGAGCTTCGTGGTGTGGGACCCGGTGGAATTCGCTAGAGTCGTGCTACCTAGGAATTTCAAGCACAACAATTTCTCCAGTTTTGTTCGGCAACTCAATACTTATCTGATGAAGTTTTTTGTACTAACATTGCGGCCATGAGTATATAAGTGAGGTTGCTGGAATTTGGTTGGTGCATTCTTTAAATATTTGATAATTTGCACGGGATTATGTAATGGAGTGTATCTGATTTTATGTATTTGTACTAAGTTAATTTTTGTAATTTTTCTGTCAATGTAATTGTAAAGCTTTCAGTTGTTTCTTAGTGAATTATAATGGGTTTCTGGGTTACCTTAGAGTTGTAATGTTGACTTGTTGGGGCATAATAGTCTCTGTAGGGCTTGTCTCCTCTATGAAATGATTACTGTAGATTTTAGAAAACCATAAGGGTTCTGCCTTCAGGTGATAGATAGTACCTGAGGTTTTGATCTTCATACTGCCAACAAAAGGACATCCTTCCCCTCTGAGCAAAGTATTGGAGCGGGGAGATACATCTTGTGCAGGTAGAATTTAAGGTTCTGGTGGAGCATTTTATTCCTATTTGACAGTGTGAAGACTCTTTATTTTCTGCATGTAATGTAAATCCATGGCTGTGACATTTTTCTTATGCAAATCAACTCTTTTGCGTAAAAATTGACAGCTTTTATGTTTTCT
