# Supplementary material for: Flower Bud Transcriptome Analysis of Sapium sebiferum (Linn.) Roxb. and Primary Investigation of Drought Induced Flowering: Pathway Construction and G-Quadruplex Prediction Based on Transcriptome
Source: PLoS One. 2015 Mar 4;10(3):e0118479. doi: 10.1371/journal.pone.0118479 (PMC4349590; doi:10.1371/journal.pone.0118479)
Supplement: S2 Text — (DOCX) [file pone.0118479.s008.docx]

Table 1 Primer sequences used in semi-quantitative RT-PCR

| Primer | Oligonucleotide |
| --- | --- |
| GA1-f | 5’- GCTGGATTACTGTTACTTTTCTTGT -3’ |
| GA1-r | 5’- GCTGTAAATATGATCTCAACTTGCT -3’ |
| CRY2-f | 5’- AAGAAGAGAACTCAATAGGGAAAGA -3’ |
| CRY2-r | 5’- ATCACCCTTATTCTATTGTGTATCC -3’ |
| AP2-f | 5’- AAGAAGATGGCAGCGGCGAGAGAGG -3’ |
| AP2-r | 5’- TCCGGTAGTAGGTGACACCACGATA -3’ |
| LFY -f | 5’- GCCTTGATTACCTCTTCCATCTTTA -3’ |
| LFY -r | 5’- ATAGTGCCGCATTTTGGGTTTG -3’ |
| SEBIN -f | 5’- ATGTGGTTGCTTATCGTGTAGGAA -3’ |
| SEBIN -r | 5’- GATATATTTGAATCGTGCCGCC -3’ |
| PRXII25514-f | 5’- TCCGATTTCCGCTGGTGATA -3’ |
| PRXII25514-r | 5’- GAGCCTTTAGGATTTCATCAACA -3’ |
| PRXII39562-f | 5’- CTCCCTCTCAAACACCAAACCA -3’ |
| PRXII39562-r | 5’- TTCCATCGGATAAAAGCAGCA -3’ |
| GRX50435-f | 5’- ATGGCGGCGATGAGAATGAG -3’ |
| GRX50435-r | 5’- TGCTTCCCATTTATGAAAACCTGTG -3’ |
| GRXII29453-f | 5’- ATGGCGACGACGAAGGCTAAAG -3’ |
| GRXII29453-r | 5’- AAGACATTTGGCACAGTTCGTTG -3’ |
| ACTIN-f | 5’- CCAGAAGTCCTGTTCCAGCCAT -3’ |
| ACTIN-r | 5’- TGCCAGGGAACATAGTGGAACC -3’ |

f represents forward primer and r represents reverse primer.
